# Components of Selection in the Evolution of the Influenza Virus: Linkage Effects Beat Inherent Selection

**DOI:** 10.1371/journal.ppat.1003091

**Published:** 2012-12-27

**Authors:** Christopher J. R. Illingworth, Ville Mustonen

**Affiliations:** Wellcome Trust Sanger Institute, Hinxton, Cambridge, United Kingdom; University of California San Francisco, United States of America

## Abstract

The influenza virus is an important human pathogen, with a rapid rate of evolution in the human population. The rate of homologous recombination within genes of influenza is essentially zero. As such, where two alleles within the same gene are in linkage disequilibrium, interference between alleles will occur, whereby selection acting upon one allele has an influence upon the frequency of the other. We here measured the relative importance of selection and interference effects upon the evolution of influenza. We considered time-resolved allele frequency data from the global evolutionary history of the haemagglutinin gene of human influenza A/H3N2, conducting an in-depth analysis of sequences collected since 1996. Using a model that accounts for selection-caused interference between alleles in linkage disequilibrium, we estimated the inherent selective benefit of individual polymorphisms in the viral population. These inherent selection coefficients were in turn used to calculate the total selective effect of interference acting upon each polymorphism, considering the effect of the initial background upon which a mutation arose, and the subsequent effect of interference from other alleles that were under selection. Viewing events in retrospect, we estimated the influence of each of these components in determining whether a mutant allele eventually fixed or died in the global viral population. Our inherent selection coefficients, when combined across different regions of the protein, were consistent with previous measurements of dN/dS for the same system. Alleles going on to fix in the global population tended to be under more positive selection, to arise on more beneficial backgrounds, and to avoid strong negative interference from other alleles under selection. However, on average, the fate of a polymorphism was determined more by the combined influence of interference effects than by its inherent selection coefficient.

## Author Summary

Success in life is the product of many factors. Inherent ability often underlies great achievement. But other factors may play their part. The circumstances a child is born into may help or hinder his or her progress. Later events also have their effect; a life may be influenced by a lucky break, or an unforeseen disaster. In this work, we examine the factors underlying success for mutations in the HA gene of human influenza virus A/H3N2, defining success as the attainment of a high frequency in the global population. We examined the history of the gene from 1968 until 2010. For each observed mutation, a mathematical model was used to estimate the inherent benefit or disadvantage it conferred to the virus. We calculated the advantageousness or otherwise of the background upon which it arose, and the subsequent effect of interference from other mutations under selection. We found that successful mutations tended to have an advantageous background, and were subsequently fortunate in avoiding negative events throughout their lifetime. Beneficial mutations were more likely to be successful. But a mutation's chances of success were influenced more by circumstances of birth and subsequent events, than by its inherent effect on the virus.

## Introduction

The influenza A virus is an important human pathogen, infecting between 5 and 15% of the global population each year [1]. Infection by influenza leads to strain-specific immunity in the human population, driving a rapid process of adaptation in the virus [2], particularly in the viral surface proteins haemagglutinin (HA) and neuraminidase (NA) [3].

Influenza evolves according to two distinct processes. Firstly, variation within gene sequences arises through a high mutation rate [4], the resulting mutations being subject to selection pressure. For HA and NA, the host immune system is a key driver behind selection [5]–[8], but selection can also arise from interactions with other viral genes [9], stability of the protein structure [10], adaptation to new species [11], drug resistance [12], and, in HA, glycosylation [13]. In a second process, known as reassortment, genes from viruses co-infecting a host combine to produce novel viral strains [14].

Many previous studies have measured the importance of selection in the evolution of influenza. Constructing a dendrogram from viral sequences, and evaluating occurrences of synonymous and non-synonymous substitutions, the ratio dN/dS has been used to identify positive selection, both at given loci, and in the HA gene as a whole [15]–[17]. Other attempts to measure selection have looked for changes in frequencies, or in the distribution of frequencies, of nucleotides or amino acids, or rates of substitution between them, in individual loci, or gene-wide [18]–[22]. Each of these methods has produced estimates of selection based upon sets of multiple substitutionary events, either within single loci, or across a set of loci within a gene, rather than by considering single polymorphisms one by one.

Homologous recombination in influenza is extremely rare, if not entirely non-existent [23], [24]. As such, within the viral population, interference effects, by which selection on one allele affects the frequency of another, are likely to occur [25]–[28]. On the basis that mutations observed at antigenic sites are more likely to be fixed than those at non-antigenic sites, it has been argued that the effects of hitchhiking in influenza are small [29]. However, evidence supporting the existence of clonal interference has been identified from the timings of fixation events in the HA gene, and from the inferred number of co-existent strongly beneficial mutations [30].

Here, we consider the evolution of the HA gene of the human influenza A/H3N2 strain since its emergence in 1968. H3N2 evolves more rapidly than H1N1 [3], having acquired multiple changes in amino acids over time. While reassortment between genes has occurred in this strain [31], the HA sequences represent a consistent strand of evolution, described by a large number of sequences [32]. We converted sequences of the HA gene into time-resolved measurements of allele frequencies at polymorphic loci, using these data to infer the strength of selection acting upon each allele.

**Figure 1:**
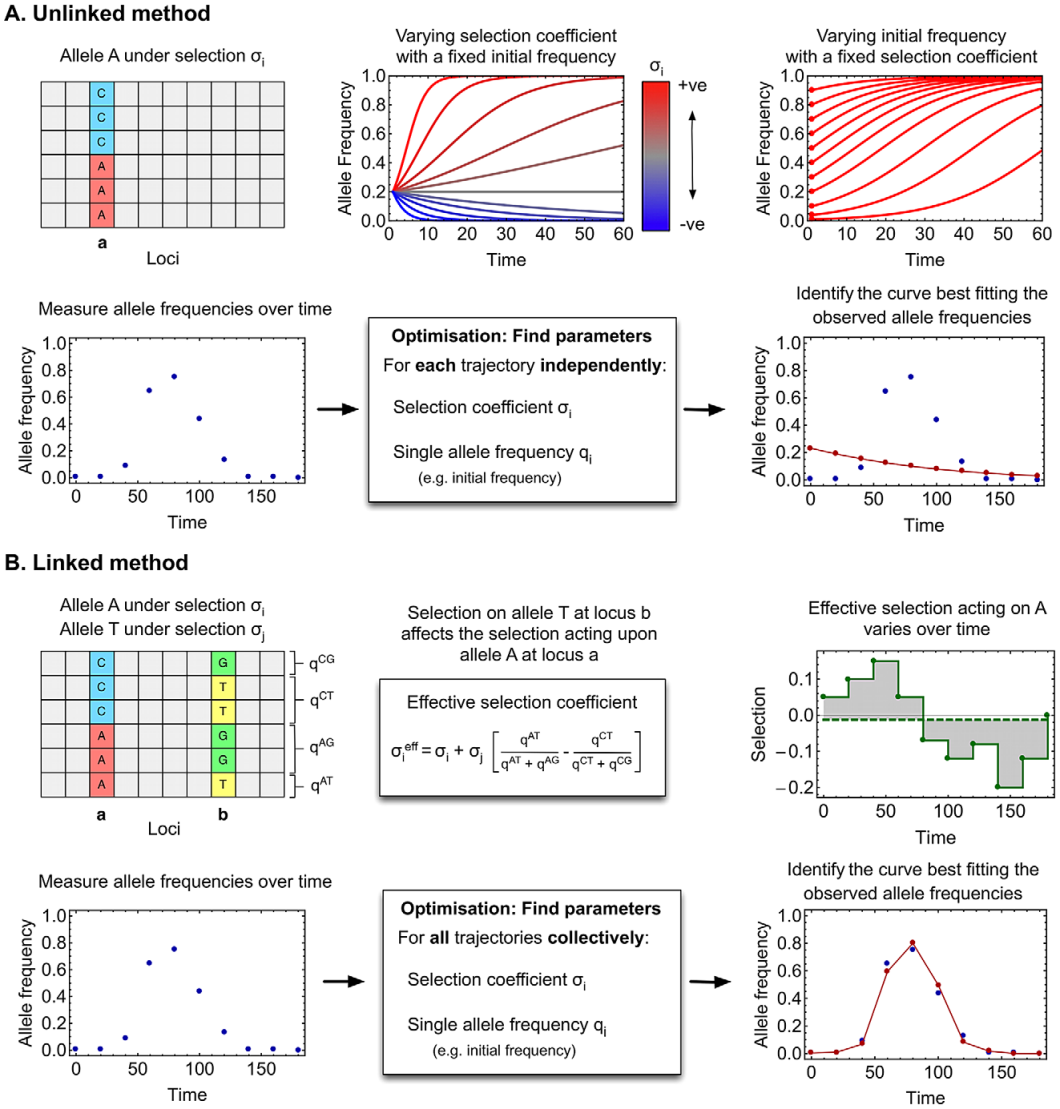
Outline of methods for inferring selection. (**A**) **Unlinked method** Above: Polymorphisms are considered in isolation. Two parameters are fitted to describe the evolution of the polymorphism over time, a selection coefficient 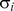, and a single allele frequency 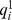. Below: Application of the method. Allele frequencies are measured over time (blue dots). The parameters are learnt independently of changes in other allele frequencies. Parameters which best reproduce the observed frequencies, under a likelihood model, are found (model frequencies shown in red). (**B**) **Linked method** Above: Polymorphisms are considered together, with two-locus haplotype frequencies being calculated from sequence data. Under an additive assumption, the effective selection on the A allele at locus 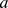 depends on selection at locus 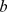, according to the equation shown (middle). Here only two polymorphic loci are illustrated; in reality selection at multiple polymorphic loci would influence the evolution of A. The effective selection 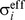 acting on A is time-dependent. It is re-calculated at each sampling point (green dots), and assumed to remain constant between sampling points (green line). The green dotted line shows the inherent selection 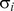. Below: The linked method is applied similarly to the unlinked method. However, all parameters are learnt simultaneously, selection on one allele potentially affecting the evolution of many others. Accounting for interference between alleles more faithfully reproduces the observed allele frequencies. Frequencies and parameters shown here are illustrative.

Inference of selection coefficients was carried out using adapted forms of two methods, described in a previous publication [33], and illustrated in Figure 1. Collected frequencies of an allele, from its first observation until its death or fixation, are referred to as the allele trajectory. Firstly, an “unlinked” method, representing a minimal model for inferring selection, was applied. The selection acting upon an allele was measured in terms of a selection coefficient 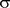, equal to the difference in Malthusian fitness between it, and the wild-type allele at the same locus. For each trajectory 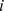, a single selection coefficient, 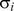, was learnt, along with a single frequency, 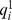, for some time-point within the trajectory (see Methods), so as to maximise the fit between the model, and the observed trajectory frequencies. Secondly, a “linked” model was applied, which used observed two-locus haplotype frequencies to account for interference effects between trajectories.

In the linked model, for each trajectory 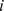, two selection coefficients were considered. The inherent selection coefficient, 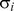, again denotes the inherent advantage or disadvantage conveyed by the allele, relative to the wild-type allele, to individuals which possess it. The effective selection coefficient, 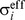, by contrast, denotes the mean total advantage or disadvantage experienced by individuals with that allele, when selection effects acting upon alleles at all polymorphic loci are considered. This effective selection coefficient is time-dependent, and is written 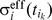, where 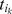 is the 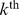 time-point of the trajectory 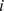. Given a set of selection coefficients for all trajectories, 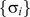, our method calculates values of 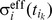 for each trajectory. Under the model, allele frequencies at each locus change deterministically according to these effective selection coefficients. Selection coefficients, 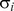, and allele frequencies, 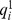, were optimised to give the maximum likelihood fit between the model, and the observed allele frequencies.

Our “linked” model represents a minimal model of selection accounting for interference effects; while it is more complex than the unlinked model, it has no additional complexity, in the sense of additional parameters to be learnt. Rather, the additional component of the model derives from the underlying sequence data. Two-locus haplotype frequencies are measured at each time-point. Next, if some allele 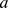 is under selection, and is in linkage disequilibrium with another allele 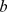, selection acting on 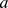 changes the frequency of the allele 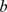 in accordance with that linkage disequilibrium. Due to its minimal nature, our linked model has some limitations. For example, the inherent selection coefficients, 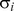, do not change with time. Polymorphic alleles were assumed to interact according to a model of additive fitness. Further, to simplify the calculation, synonymous trajectories were assumed to have zero inherent selection, moving only via linkage disequilibrium with non-synonymous trajectories. Limitations of the model are discussed later in the text.

Application of our model gave an insight into the processes driving the evolution of influenza. Recent theoretical studies of the evolution of asexual populations have underlined the importance of the background upon which polymorphisms arise, and of interference from other polymorphisms under selection [34], [35]. Experimental work has shown that, in a non-recombinant population, the background upon which a mutation lands can have a significant impact on its eventual fate [36]. On the basis of our inferences, we measured the importance of inherent selection, the initial genetic background, and subsequent interference effects, for the eventual fate of a polymorphism. Our results suggest that background effects, subsequent interference, and inherent selection are of similar importance for determining the fate of polymorphisms in the HA gene of human influenza A/H3N2. The combined effect of interference outweighs that of inherent selection.

## Results

### Summary statistics

A total of 3327 complete sequences of the HA gene of human influenza A/H3N2 were obtained from the NCBI influenza virus resource [32]. Trajectories reaching a frequency of at least 2.5% were included in the analysis (for discussion of this choice see Supporting Information), giving a total of 1638 trajectories (655 non-synonymous, 983 synonymous), spanning 256 time-points.

In the unlinked method, a selection coefficient and a single allele frequency were learnt for each trajectory, so as to obtain the maximum likelihood fit between the model, and the observed allele frequencies. In the linked method, synonymous trajectories were assumed to evolve under zero inherent selection, so for these, only an allele frequency was inferred. In the application of the linked method, pairwise interactions between all simultaneously polymorphic trajectories were accounted for; a mean of 4939 such pairs existed at any one given time-point.

One limitation in the inference arose from a lack of available sequences at early time points (see Supporting Information). As a result only polymorphisms which arose from 1996 onwards were included in the final analysis (spanning 84 time-points). The statistics that follow encompass 622 trajectories, of which 238 were non-synonymous, representing polymorphisms across 442 nucleotide positions (at 339 amino acid residues) in the HA gene. For these trajectories each observed allele frequency was calculated from a sample of mean size 202 sequences (range 57 to 449).

### Linked and unlinked models of evolution

**Figure 2:**
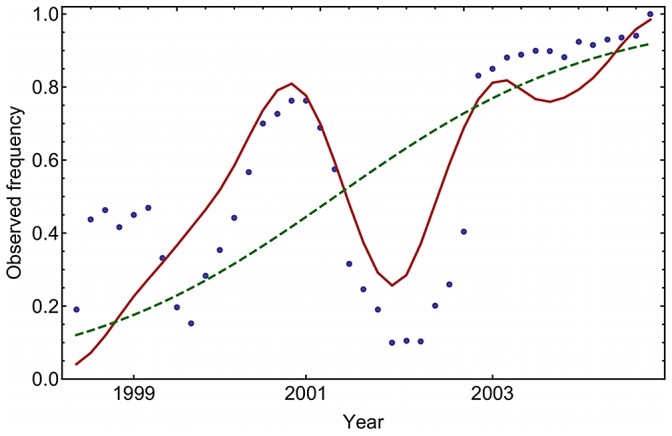
Modelling linkage disequilibrium allows the fitting of complex trajectories. Inferences for a trajectory describing a polymorphism from ATT to AAT in locus 161. The trajectory inferred by the “linked” method (solid red line) gives a substantially better fit to the observed trajectory (blue dots), than does the trajectory inferred by the “unlinked” method (dashed green line). Including the effect of linkage disequilibrium between alleles gives time-dependent effective selection coefficients 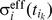 that capture key features of the observed trajectory.

Including the effects of linkage disequilibrium between polymorphisms allowed the linked model to capture much of the complex behaviour of the observed trajectories. Relative to the unlinked approach, the model gave a substantially better fit to the observed trajectories (Figure 2 gives one example). In the unlinked model, the inference of selection coefficients for synonymous trajectories required, for the trajectories from 1996 onwards, an additional 384 parameters to be learnt in the optimisation. Nevertheless, the unlinked model gave a substantially worse fit to the data. Measured across more than 7000 observed frequencies, the mean absolute difference between inferred and observed allele frequencies was larger, at 0.09, for the unlinked model, than the equivalent value of 0.05 from the linked model. Where linkage disequilibrium is ignored, inferred frequencies are restricted to change according to a model of constant selection, giving a monotonically increasing or decreasing trajectory. Many trajectories encompassed both rising and falling allele frequencies, a phenomenon which, in a deterministic framework, requires a model of variable selection. Plots of all trajectories, together with model fits, are given in Supporting Information. We now discuss results obtained using the linked method.

### Inherent selection and interference effects

**Figure 3:**
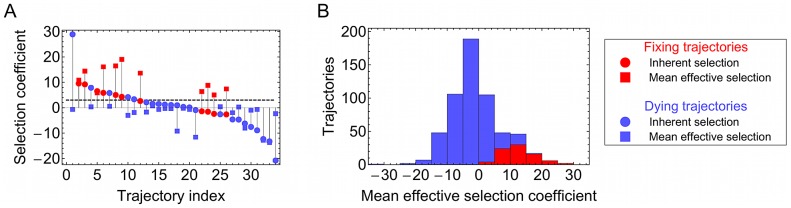
Inferred and mean effective selection coefficients show substantial interference effects. (**A**) **a snap-shot**:Values of 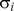 (circles) and 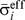 (squares) inferred for trajectories which were polymorphic in the sample taken on 29th October 2002, ordered by 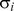. Substantial differences are seen between the inferred and mean effective selection coefficients, demonstrating significant interference effects. Interference is seen to have a substantial effect on the fate of polymorphisms; all trajectories which proceed to fixation (red) have 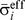 above the dashed horizontal line, while all trajectories which eventually die out (blue) have 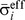 below the line. (**B**) **all data since 1996**: Histogram of 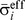 values for trajectories, those which died being shown in blue and those that proceeded to fixation being shown in red.

Our model suggested interference effects to be of substantial importance in determining the eventual fixation or death of mutations in the influenza virus. For each trajectory 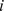, a measure, 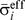 of the total selection acting upon the mutant allele (including both inherent effects and those arising from linkage disequilibrium) was calculated, as the mean value over time of the effective selection coefficient:
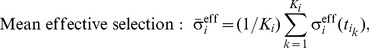
(1)where 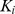 is the length of trajectory 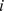. Considering sets of polymorphic alleles at given time-points, substantial differences between 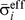 and 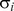 were seen (see for example Figure 3A). Both clonal interference and genetic hitchhiking were evident, with mutations under strong positive inherent selection, but negative interference, dying out, and mutations with lesser inherent selection, but experiencing positive interference, reaching fixation. Both in the examples shown, and across all trajectories (Figure 3B), the statistic 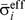, inferred after the event, discriminated strongly between mutations that fixed and those which died out.

### Components of selection

Derivative components of selection, associated with interference effects, were calculated for each of the trajectories. Firstly, we derived a measure of the mean effect, over time, of interference acting upon each trajectory. We note that the mean effective selection coefficient, 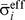, calculated above, combines the inherent selection acting on a trajectory, 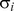, plus interference effects from other trajectories. We calculated a measure, 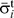, of the mean interference acting on a trajectory 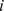 by taking the difference of the mean effective and inherent selective effects:
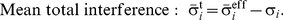
(2)This interference term can be divided into two parts, representing the effects of interference in the initial and later parts of the history of a polymorphism. Considering these in turn, any given mutation observed in the population is first seen in the context of a specific sequence background. Within the global viral population at any one time, non-synonymous polymorphisms at frequencies considered by our method (reaching at least 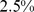) exist at multiple loci. The sequence(s) upon which a mutation arises contain specific alleles at these loci, this background conferring an initial fitness effect. We estimated this background fitness for a trajectory 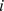, denoted 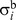, as the mean interference over the period before the trajectory reached a frequency of 2.5%:
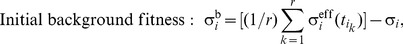
(3)where 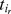 denotes the first time at which the allele of trajectory 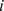 was observed at a frequency of 2.5% or greater. Subsequently, across the lifetime of a trajectory, its evolution is affected by the arrival of new mutations under selection. Changes also occur in the effect of its background; as the mean fitness of the population increases, any given background becomes relatively less beneficial. We measured the combined effect of these factors, terming it the post-emergence interference, 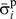. This statistic was calculated by subtracting the initial background effect from the total interference:
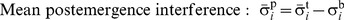
(4)We note that each of the above components of selection are derived from the inferred selection coefficients 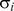, rather than being learnt independently from the data.

Across trajectories, the magnitude of the effect of interference was generally greater than that of inherent selection. Measured for non-synonymous trajectories, the mean absolute effect of interference acting on a polymorphism at a given time was larger, at 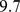, than the mean absolute inherent selection of 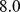 (selection is described throughout in units of 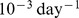). Simple characterisation of interference in terms of a few large effects was difficult; at any given time, a mean of 15 other trajectories made an absolute contribution of more than 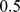 to the effective selection coefficient of a polymorphism, from a total of 29 trajectories contributing more than 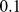.

**Figure 4:**
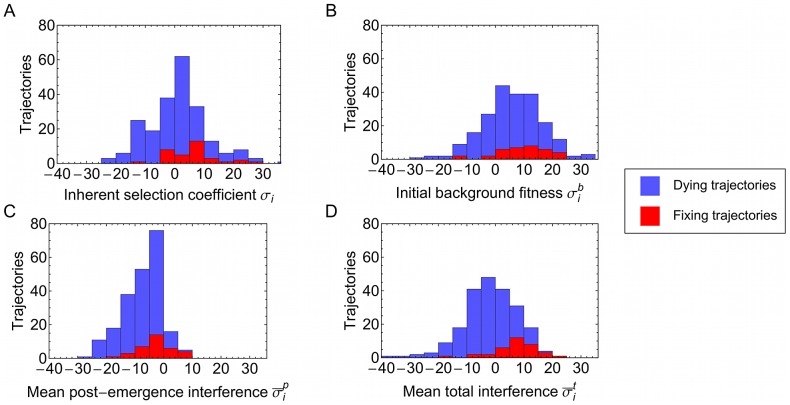
Components of selection: breakdown of selection and interference effects. Histograms show the total distribution of values obtained for non-synonymous trajectories, trajectories which died shown in blue and which proceeded to fixation in red. (**A**) Inherent selection coefficients 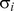; (**B**) Initial background fitness 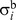; (**C**) Mean post-emergence interference 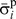; (**D**) Mean total interference 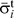. Distributions are calculated across a total of 218 trajectories. Fixing trajectories tended to appear on more positive backgrounds, and subsequently avoided strong negative interference.

To examine selective effects in more detail, we calculated distributions of each statistic for trajectories that eventually fixed, and for those that died out (Figure 4 shows data for non-synonymous trajectories). In each case, a bias can be seen, trajectories proceeding to fixation having generally more positive coefficients of selection and interference. Different components of selection biased the fates of trajectories in different ways. For example, the initial background for observed trajectories was generally positive, being more so for fixing trajectories, while the mean post-emergence interference was predominantly negative. Fixing mutations tended to arise in good backgrounds, and were subsequently fortunate in avoiding strong negative interference.

**Figure 5:**
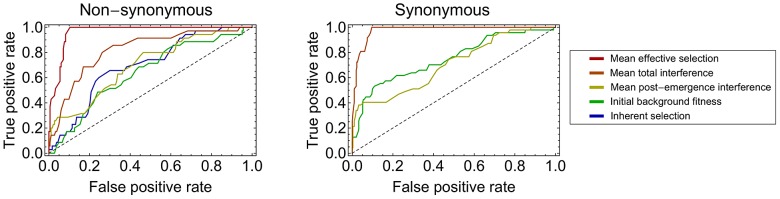
Ability of retrospective measurements of selection to divide fixing from non-fixing trajectories. Values are shown for non-synonymous and synonymous polymorphisms, representing statistics for the inherent selection coefficient, 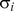 (blue), the initial background fitness, 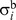 (green), the mean post-emergence interference, 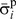 (yellow), the total mean interference, 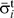 (orange), and the mean effective selection coefficient, 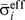 (red) of a trajectory. The black dotted line indicates a null expectation, describing a hypothetical method with no power to divide fixing from non-fixing trajectories. For synonymous polymorphisms, inherent selection coefficients were set to zero. As such, the measures for inherent and mean effective selection coefficients are omitted, the former being equal by default to the null expectation, and the latter being equal to the total mean interference.

In order to quantify the importance of each of the components of selection for the fate of polymorphisms, we measured the accuracy with which each component, considered in retrospect, separated trajectories which reached fixation from those that died out. Across non-synonymous trajectories, the inherent selection coefficient showed some ability to perform this task, with a calculated accuracy of 0.70 (Figure 5). Both the initial background fitness, and the post-emergence interference, performed similarly well, with measured accuracies of 0.64 and 0.70. When interference effects were combined, the accuracy achieved was greater than that for inherent selection, returning a value of 0.82.

Across synonymous trajectories, the inherent selection coefficient was set by default to zero, such that the effect of any inherent selection could not be measured. However, for these trajectories, the accuracies of the initial background and post-emergence interference were again roughly equivalent, at 0.75 and 0.71 respectively. The evolution of synonymous trajectories was well-explained purely in terms of interference from non-synonymous polymorphisms under selection; absolute differences between observed and inferred frequencies were no larger than those for non-synonymous trajectories.

### Selection across the HA gene

Measuring the relative strengths of selection acting upon different parts of the HA protein, we obtained results equivalent to those previously obtained from measurements of dN/dS, the ratio between rates of non-synonymous and synonymous mutations. We derived a region-wide measure of selection by calculating the mean of the inherent selection coefficients inferred for non-synonymous trajectories at loci in that region. This measure was then calculated for trajectories in the HA1 and HA2 segments of the protein, and for both epitope and non-epitope loci. Substitutions in the influenza phylogenetic tree can be divided into those occurring on trunk and side branches [37]; to parallel this, we calculated separate measurements for trajectories which fixed in the global population (c.f. trunk substitutions), and for those which died out (c.f. side-branch substitutions). A previous calculation performed on substitutions in a tree of sequences from the human influenza A/H3N2 strain between 1994 and 2005 found dN/dS ratios fitting the order epitope 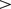
HA1
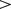
HA2, with ratios in each case higher for substitutions in trunk branches than in non-trunk branches [17]. Our measurement replicated this finding across corresponding sets of trajectories (Table 1).

**Table 1:** Mean inherent selection coefficients inferred by the method.

Protein sites	Mean inherent selection		
	All trajectories	Fixing trajectories	Dying trajectories
HA	1.12	6.80	0.04
HA1	1.33	7.71	0.10
HA2	0.18	2.38	−0.22
Epitope	2.29	8.53	0.54
Non-epitope	−0.15	2.47	−0.43

Mean values of 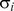 inferred for non-synonymous trajectories within the HA1 and HA2 parts of the gene, and for epitope and non-epitope residues, considering both fixing and dying trajectories.

The levels of significance underlying our results were calculated by applying a Kolmogorov-Smirnov test to to the inferred distributions of selection coefficients; differences in inferred selection coefficients between epitope and non-epitope trajectories, and between the subclasses of fixed epitope and fixed non-epitope trajectories were each significant (p = 0.014 and p = 0.021 respectively). A significant difference was seen between selection coefficients at epitope and HA2 trajectories (p = 0.030), though differences between trajectories in HA1 and HA2 were not significant at the 95% level. In our calculation, epitope loci were identified according to the alignment of Wolf et al. [17].

The difference between epitope and non-epitope loci was also seen via measurements of fitness flux, a statistic quantifying the total amount of adaptation at a locus [38]. Across trajectories reaching fixation or death, the total fitness flux for a trajectory 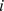 is given simply by 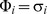 if the trajectory fixed, and 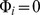 if the trajectory died. On the basis of our inferred selection coefficients, the total fitness flux across trajectories at epitope loci was approximately 8.6 times larger than the flux across trajectories at non-epitope loci. While both beneficial and deleterious mutations were observed in all parts of the gene, this result implies that events at epitope loci are of substantial importance for the adaptation of the virus.

Regions in which beneficial mutations were identified correlated with those under lesser evolutionary constraint. The extent of evolutionary constraint was measured by examining the number of non-synonymous polymorphisms observed across different sets of multiple loci. Supposing a constant mutation rate across the gene, and lack of any constraint, two regions each comprising a similar proportion of the gene would be expected to harbour a similar proportion of non-synonymous mutations. In regions with an increased constraint on mutations, as might arise from requirements of protein function or structural stability, a greater number of these mutations would be strongly deleterious, leading to a relative under-representation of non-synonymous trajectories from that region. In our data, the HA2 region, and, to a lesser extent, the non-epitope region of HA1, were each underrepresented for observed polymorphisms and trajectories, while the HA1 region as a whole, and in particular, epitope loci, were overrepresented. This pattern was exaggerated for trajectories identified to be under positive selection (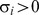), and further so for trajectories with 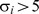 (Table 2), suggesting that sites experiencing greater numbers of beneficial mutations, were also sites of lesser evolutionary constraint. This result corresponds to the HA protein structure, in which epitope residues tend to be in regions more exposed regions of the protein [39].

**Table 2:** Over- and under-representation of trajectories in different regions of HA.

Protein sites	Number of events relative to size			
	All polymorphisms	All trajectories	Trajectories ( 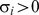 )	Trajectories ( 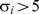 )
HA1	1.29***	1.40***	1.46***	1.50***
HA2	0.59***	0.43***	0.36***	0.30***
Epitope	2.05***	2.40***	2.60***	2.97***
HA1 non-epitope	0.84***	0.81*	0.77*	0.63**

Proportion of trajectories occurring in different regions of HA. Values are expressed relative to the size of each region, measured by number of residues. The set of all polymorphisms includes trajectories excluded from the analysis (i.e. with maximum frequency less than 2.5%). Deviations from the expected value of 1 are labelled according to their significance:

* p
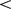
0.05;

** p
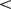
0.01;

*** p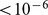.

## Discussion

The problem of inferring the selection acting at every polymorphism within the history of the human influenza A/H3N2 HA gene represents a significant challenge. Here we have examined the role of interference, as opposed to inherent selection, in determining the eventual fixation or death of polymorphisms. We identified, first, that interference is of great importance in understanding the evolution of influenza. While having no more parameters than the unlinked model, our linked inference model produced trajectories that substantially better fitted observed allele frequencies over time. Next, examining statistics of selection from our linked model, we inferred that, for non-synonymous polymorphisms, the total effect of interference acting on a polymorphism was, in the mean, more significant for its fate than was its inherent selection coefficient. With the inherent selection acting on synonymous polymorphisms expected to be substantially weaker than that acting on non-synonymous polymorphisms (see discussion later), our result is likely to hold for polymorphisms in the HA gene in as a whole.

For both non-synonymous and synonymous events, the effect of interference was split roughly equally between background effects, and subsequent interference. While we do not make specific estimates of population size, mutation rate, or the distribution of fitness effects (we infer selection coefficients only for polymorphisms which are observed at above a given frequency), the key role we observe for background and interference effects is in line with mathematical expectations for an asexual system with high rates of mutation and positive selection [34], [35].

While we replicate results previously obtained using a measure of dN/dS, our approach to inferring selection is substantially different. For example, whereas dN/dS comprises a statistic, calculated across multiple events, we attempt to infer a selection coefficient for each individual polymorphism. Further differences lie in our use of time-resolved frequency data; rather than inferring a phylogeny we consider sequences in terms of their allele and haplotype frequencies. A trajectory-based approach may differ from a phylogeny in terms of the number of times a mutation is observed; multiple identical substitutions in different parts of a tree might comprise one or multiple trajectories. However, several features in the H3N2 HA gene that have been identified to be under selection, including repeated mutations at a site, selective sweeps, and highly polymorphic sites [20], were visible in our set of allele trajectories. We note that, where sequences are taken from a single population, the interpretation of values of dN/dS is not always straightforward [40].

Our inference of strong interference effects has implications for the evolution of influenza. Firstly, interference between beneficial mutations slows down the overall rate of adaptation [25]. The lack of recombination within genes of influenza means that beneficial mutations compete against each other for success. A mutation causing the virus to escape the immunity of the host population might be outcompeted by another. The most inherently advantageous mutations are not guaranteed to fix. Further, interference makes the prediction of viral evolution more difficult. Identifying the most beneficial mutations at a given time does not necessarily identify those that will go on to fixation. A polymorphism that was growing in frequency might be killed off by the emergence of another, more beneficial mutation. A polymorphism that existed at low frequency, with no apparent benefit for the virus, might, through hitchhiking, reach fixation.

### Model limitations

As noted above, our linked model represents a minimal approach for inferring selection while accounting for interference effects. While our model provides strong evidence of the importance of interference effects, it does not account for the full biological complexity of influenza, making a number of assumptions and approximations.

Firstly, synonymous mutations were assumed to have zero inherent selection coefficient, synonymous trajectories changing their frequency purely by the effect of linkage disequilibrium with non-synonymous trajectories. Such an assumption is not unique to our method, reflecting similar assumptions made, for instance, in the measurement of dN/dS. However, the identification in HA of some selection for RNA secondary structure [41] and of long-term changes in codon preference [19] each suggest the possibility that some synonymous changes are non-neutral. This shortcoming in our method is unlikely to affect our central result, of the importance of interference effects. While individual synonymous mutations may be under significant selection, the magnitude of selection, considered across groups of trajectories, is likely to be substantially lower for synonymous than non-synonymous mutations. As such, any errors in our statistics of selection arising from this assumption are likely to be small when considered across the set of all trajectories.

Secondly, in our use of a deterministic model for the evolution of trajectories, we do not take into account genetic drift, a process, independent of selection, in which the transmission of alleles from one generation to the next introduces noise into the allele frequencies. (Within our framework, the frequency of an allele evolving under zero selection would remain constant.) We believe that the effect of this omission is also likely to be small. Considering alleles at intermediate frequencies, we note that genetic drift is a slow process. In influenza it has been estimated that the expected time for an allele to fix under drift alone is of the order of 200 years, far longer than the typical length of a trajectory [29], implying that frequency changes are driven much more by selection than by drift. As evidenced by antigenic drift, strongly selected alleles arise frequently [6], and, due to the lack of recombination, are likely to remain in linkage disequilibrium with other alleles at polymorphic loci. Interference arising from these strongly selected alleles would mean that changes in the frequencies of neutral alleles would also be dominated by selection [34]. Considering alleles at lower frequencies, the influence of selection on an allele is overtaken by that of drift below a frequency of 1/N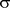, where 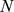 is the population size and 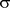 the magnitude of selection acting on the allele [42]. In recent work, an estimate has been made that in H3N2 influenza this threshold is close to 1% [30]. Across our observed trajectories, one out of 12 observed frequencies were below this threshold. If this threshold estimate is correct, our exclusion of trajectories with minimal frequencies less than 2.5% ensures that the majority of our trajectories are influenced more by selection than drift for the majority of their lifetimes.

Thirdly, the inferences we make are drawn on the basis of limited data. While we filter trajectories to consider only those for which we had enough data to derive consistent results, studies of simulated systems suggest that a larger sample would lead to more accurate inference. Consideration of lower frequency events may affect our estimate of the relative importance of the initial background and subsequent interference on a polymorphism; measuring at lower frequencies, more deleterious mutations, and mutations on more deleterious backgrounds, would likely be seen.

We further note that, while detecting selection, our method does not infer reasons for this selection. The molecular basis for the evolution of influenza is complex, involving multiple host-virus interaction processes, and the biophysical properties of the viral proteins. Each of these processes may result in changes in allele frequencies, and their mean effect can potentially be recaptured via inference. However, assignment of selection to specific physical effects would require substantial further work. Our approach is not well-suited to identifying the magnitude of selection arising from evolutionary constraints, such as protein structural stability; where an allele is under strong purifying selection, by its nature it is not likely to be observed at frequencies high enough for selection to be inferred.

Extensions to the linked model could be made to include the possibility of time-dependent inherent selection acting on a given allele, or of non-additive models of interference between trajectories. Either of these would add to the complexity of the model, and would require care in implementation.

Time-dependent inherent selection in influenza might arise through the acquisition of strain-specific immunity among the human population to an antigenic change in the virus [43], [44]. A mutation causing a novel antigenic type might initially be at a selective advantage within the viral population. However, as people encountered the new antigenic type, and acquired immunity, this selective advantage would decrease. Variable selection corresponding to antigenic type has been included in models of influenza evolution by mapping changes in genetic sequence to changes in antigenic type [2], [45], [46]. Time-dependent selection could be incorporated into the model presented here either with the incorporation of antigenic data, or simply by allowing inherent selection coefficients to vary over time. However, we note, first of all, that time-dependent selection would not remove the role of interference. Linkage disequilibrium between alleles implies that the evolution of one allele is affected by any non-neutral selection acting on the other, whether or not that selection is time-dependent. Secondly, any adding of parameters to the model requires care to avoid over-fitting; in the limit case, allowing the inherent selection on each allele to vary between each time point would result in a perfect fit to the observations by default. The inherent selection coefficients inferred here are best interpreted as mean values over time.

Epistasis (non-additive selective effects) between alleles has been detected in the HA and NA genes of influenza by considering patterns of ordered substitution events [47]. Full inclusion of epistasis into a model, however, is difficult, due to the rapid inflation of the number of parameters required. With the addition of a substantial number of parameters, epistatic effects between pairs of mutations could potentially be modelled. However, there is no guarantee that epistasis operates purely on this level; interactions between three, four, or more mutations may also play a role in viral fitness. Our model includes epistatic effects where all but one of the mutations in question are fixed; repeated instances of identical mutations are assigned independent selection coefficients. We note that, under certain circumstances, epistasis may be irrelevant. Where two alleles are seen only in isolation in the population, and never together in combination, epistatic effects between them do not come into play. Beyond this, incorporation of epistasis into the model would be challenging, and was not attempted here, the assumption of additive fitness effects representing a first approximation.

### Interference and viral evolution

Using a minimal model accounting for interference, we have here characterised selection in the HA gene of influenza at the level of individual polymorphisms. Our approach takes into account the fact that the gene evolves as part of a haploid, asexual virus with high mutation rate, and essentially no homologous recombination, so that linkage disequilibrium between polymorphisms has a substantial impact on its evolution. Examining statistics of selection inferred for historic data, our model suggests that the combined effect of interference is more important than the effect of inherent selection for the fixation or death of a polymorphism. While many methods for inferring selection exist, we believe that it is by accounting for the specific genetic properties of systems such as influenza that progress in understanding their evolution will most rapidly be made.

## Methods

### Obtaining polymorphism data

Complete sequences for the HA gene of the subtype H3N2 of human influenza A were obtained from the NCBI influenza virus resource [32], including all sequences sampled from 1968 until 7th Feb 2011, a total of 3327 sequences. Sequence alignment of protein sequences was carried out using MUSCLE [48], corresponding RNA sequences being matched to this alignment using in-house software. Beginning at 29th June 1968, samples of sequences were taken every 60 days, each sample comprising all sequences recorded within 180 days of the sample point. Sampling in this manner grouped viral sequences taken from all seasons in both hemispheres, accounting for geographical and seasonal differences. Ambiguously dated sequences were assigned to the central day of their specified year or month of sampling.

### Calling trajectories

The frequency of a given allele was measured as the fraction of sequences within a sample having that allele at the time of measurement. Trajectories were defined as sets of observations of codon frequencies differing from the wild-type codon at their respective position. The wild-type codon for each position was initially defined according to the consensus codon from the first sequence sample; subsequent to this, a codon which reached fixation was defined as the new wild-type for that position. The identification of the point at which a trajectory has fixed or died is non-trivial. Within a single patient, the high mutation rate and large viral population size of influenza make it likely that every viable single-mutant of an infecting virus will likely exist, such that concepts of fixation and death require a qualified understanding. We regarded a trajectory as having fixed if, in our sample of the global population, we observed it at a frequency of greater than 97.5% for eight consecutive time points, and to have died if we observed a frequency of less than 2.5% for eight consecutive time points. The wild-type codon at a position was used to classify new mutations as synonymous or non-synonymous. Synonymous polymorphisms were set to have inherent selection coefficient equal to zero, changes in their frequency being inferred to arise through linkage disequilibrium with non-synonymous polymorphisms. In order to reduce the influence of noise from sampling error, and to avoid difficulties in inferring selection from very sparse data, trajectories with very low maximal frequencies (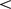
2.5%) were excluded from the optimisation (see Supporting Information).

### Inferring inherent selection coefficients

In an earlier publication we described two methods for using time-resolved data for inferring the selection acting upon a polymorphic allele [33]. For notation, we describe a set of observations of the frequency of a polymorphic allele collectively as an allele trajectory. We consider a trajectory 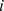, describing the evolution of a mutant allele at a two-allele locus 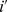, at which wild-type and mutant loci are denoted 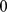 and 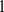 respectively (separate annotation for trajectories and loci, while superfluous here, becomes necessary in the multi-allele case). The observed frequency of the mutant allele at locus 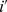 at time 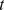 is denoted 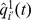. We assume that the mutant allele has a constant, trajectory-specific selection coefficient, 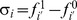, defined as the difference in Malthusian fitnesses of the two alleles at the given locus.

Our first model, referred to above as the “unlinked” model, assumes that alleles at different loci evolve independently of each other. Using the above notation, the expected frequency of the mutant allele at locus 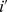 evolves according to the equation
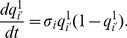
(5)This equation describes a family of trajectories, specified by the allele frequency at time 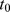:
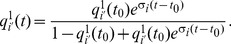
(6)In practise, the allele frequency at any time point may be used to specify curves; the central point of the trajectory, denoted 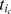, was used in our calculations. The model which best fits the observed trajectory was identified by optimising the selection coefficient and allele frequency (here 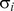 and 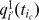) using a binomial model to fit inferred frequencies 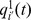 to the observed frequencies 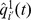.

Our second model, referred to above as the “linked” model, is essentially the same as this, but for the constraint that, if two alleles are in linkage disequilibrium, a change in the frequency of one allele, caused by selection, implies a change in the frequency of the other. At each time-point, two-locus haplotype frequencies, denoted 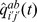 for loci 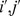 and 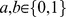, were observed and used to calculate the effect of selection at one locus on the frequency of another.

Given a trajectory 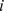 representing the evolution of the allele 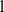 at locus 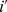, a time-dependent selection coefficient 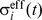 was calculated. This term describes the selection acting on the mutant allele of the trajectory given its own, or inherent selection coefficient, 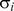, and the selection acting as a result of linkage disequilibrium with alleles at other loci, which were also under selection, at the time 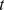. Under a model of additive selection, this can be approximated by
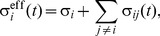
(7)where
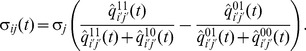
(8)The values 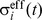 were assumed to remain constant between sampling points. Under the model, these time-dependent selection coefficents were fed into Eq. (6), to describe the evolution of the trajectory; again a fit was performed between the inferred and observed frequencies. For simplicity, we have here described the case in which there are two alleles per locus. In the adaptation of our previously published method to influenza data, changes were made to account for the process via which trajectories were sampled, and to allow independent trajectories at the same locus to have differing selection coefficients. Further changes were made to the manner in which the fixation or death of trajectories was handled, to the treatment of very low frequency trajectories and of non-polymorphic observations in the middle and ends of trajectories, to the precise use of effective selection coefficients in inferring trajectories, and to the fitting of inferred to observed trajectory frequencies. Fuller details of the method are given in Supporting information.

## Supporting Information

Text S1 Expanded description of the basic inference method. Further details of adaptations to the inference method conducted for the purposes of this study. Details of assessment of the method using simulated data. Description of the method for calling trajectories, and of an alternative method. Details of the optimisation routine. Details of the consistency of results obtained from the influenza dataset. Supporting Figures S1 to S13 are contained within the Supporting Text.
(PDF)

Figure S14 Plots of all observed and inferred trajectories inferred using the unlinked method.
(PDF)

Figure S15 Plots of all observed and inferred trajectories inferred using the linked method.
(PDF)

Figure S16 Plots of all observed and inferred trajectories inferred using the linked method with trajectories called using an alternative method.
(PDF)
