# Supplementary material for: Components of Selection in the Evolution of the Influenza Virus: Linkage Effects Beat Inherent Selection
Source: PLoS Pathog. 2012 Dec 27;8(12):e1003091. doi: 10.1371/journal.ppat.1003091 (PMC3531508; doi:10.1371/journal.ppat.1003091)
Supplement: Text S1 — Expanded description of the basic inference method. Further details of adaptations to the inference method conducted for the purposes of this study. Details of assessment of the method using simulated data. Description of the method for calling trajectories, and of an alternative method. Details of the optimisation routine. Details of the consistency of results obtained from the influenza dataset. Supporting Figures S1 to S13 are contained within the Supporting Text. (PDF) [file ppat.1003091.s001.pdf]

Components of Selection in the Evolution of the Influenza Virus: Linkage Effects Beat Inherent Selection

Christopher J. R. Illingworth and Ville Mustonen
Wellcome Trust Sanger Institute, Hinxton, Cambridge, UK
E-mail: Corresponding vm5@sanger.ac.uk; ci3@sanger.ac.uk

Supporting Information

This supporting document is divided into three sections, the first concerning the methodology applied in this work, and the second and third concerning results generated from the influenza dataset. In section 1, we outline details of the inference framework used, describing in detail changes made to the method, the principles of which were described in an earlier publication [1]. We next describe our assessment of the inference framework based upon data derived from simulated populations, with both high and low levels of sampling. Finally, we describe, in slightly more detail than given in the main text, the means by which trajectories were called from the sequence data, and describe the optimisation process used to find inherent selection coefficients. In section 2, we begin by examining the consistency of the results obtained from the method, considering results obtained within and between different optimisations. We examine the reproducibility of the key results described in the main text between optimisations, and briefly consider an alternative approach to calling trajectories. Finally, in section 3, we give details of calculations additional to those presented in the main text.

1 Descriptions and validations of our methodology

1.1 Overview of the method

We here reproduce an outline of our method, described in an earlier publication [1].

1.1.1 Observations

We begin with a set of sequences, taken from a population at discrete time points. Taking successive samples from the population, allele and two-locus haplotype frequencies are calculated at each time point. We define a trajectory to be a collection of observations of the frequency of a mutant allele (i.e. other than the wild-type) at a polymorphic locus, from the first time of observation, until the time of fixation or death. Given a trajectory i, occurring at the locus i', and observed at times $\{t_{i_k}\}$, observed allele frequencies are formally denoted $\hat{q}^a_{i'}(t_{i_k})$ for $a \in \{0, 1\}$, where $a = 1$ denotes the mutant allele, and $a = 0$ denotes the wild-type allele. (Separate annotation for trajectories and loci, while superfluous in the two-allele per locus case, becomes necessary in the general case). Observed two-locus haplotype frequencies involving mutant alleles at loci i' and j', described by the trajectories i and j, are denoted $\hat{q}^{ab}_{i'j'}(t_{i_k})$, for $a, b \in \{0, 1\}$.

1.1.2 Aside: use of shorthand notation

To improve the readability of equations, we here make use of shorthand notation. In the following section, we consider, in general, a trajectory i at the locus i'. Further, in describing the interaction with other trajectories, we generally consider a second trajectory j, at locus j'. As such, we adopt the shorthand notation t_k to refer informally to t_{i_k}, $\hat{q}^a$ to refer informally to $\hat{q}^a_{i'}$, and $\hat{q}^{ab}$ to refer informally to $\hat{q}^{ab}_{i'j'}$. Formal notation is retained in definitions, and used where necessary; the precise meaning of terms should be kept in mind.

1.1.3 Inherent selection coefficients and time-dependent selection

Suppose that trajectory i occurs at locus i', and that the wild-type and mutant alleles of trajectory i have Malthusian fitness $f^0_{i'}$ and $f^1_{i'}$ respectively. The inherent selection coefficient σ_i is then defined as $\sigma_i = f^1_{i'} - f^0_{i'}$. Assuming a model of additive selection, the selection acting on the mutant allele of trajectory i at time t_k, referred to as the effective selection coefficient $\sigma^{\text{eff}}_i(t_k)$, is given (accurate to sampling error) by

$$\sigma^{\text{eff}}_i(t_k) = \sigma_i + \sum_{j \neq i} \sigma_{ij}(t_k), \tag{1}$$

where $\sigma_{ij}(t_k)$ is the effect of selection acting on the mutant allele of trajectory i resulting from selection on the mutant allele of trajectory j:

$$\sigma_{ij}(t_k) = \sigma_j \left(\frac{\hat{q}^{11}(t_k)}{\hat{q}^{11}(t_k) + \hat{q}^{10}(t_k)} - \frac{\hat{q}^{01}(t_k)}{\hat{q}^{01}(t_k) + \hat{q}^{00}(t_k)} \right), \tag{2}$$

or alternatively,

$$\sigma_{ij}(t_k) = \sigma_j \left(\frac{\hat{q}^{11}(t_k)}{\hat{q}^{1}(t_k)} - \frac{\hat{q}^{01}(t_k)}{1 - \hat{q}^{1}(t_k)} \right), \tag{3}$$

where σ_j is the inherent selection acting upon trajectory j. These equations have a straightforward interpretation. The effective selection coefficient may be thought of as the total selection acting on the mutant allele at locus i', including both inherent selection and interference from other polymorphisms. Given additive selection, this is given by the inherent selection on the mutant allele, plus a sum of pairwise interference effects between polymorphisms (Eq. 1). The interference effect arising from a single trajectory j at locus j' is composed of two terms (Eq. 3). Firstly, haplotypes with the mutant allele at i' which also have the mutant allele at j' gain a selective benefit proportional to σ_j (the benefit being negative if $\sigma_j < 0$), the effect of this benefit on the mutant allele frequency at i' being equal to the fraction of haplotypes with the mutant allele at i' which also have the mutant allele at j'. Secondly, haplotypes with the wild-type allele at i' which have the mutant allele at j' gain a selective benefit proportional to σ_j (again being negative if $\sigma_j < 0$), the effect of this benefit on the mutant allele frequency at i' being equal to minus the fraction of haplotypes with the wild-type allele at i' which also have the mutant allele at j'.

1.1.4 Deterministic trajectories

Given time-dependent effective selection coefficients, a deterministic representation of the evolution of the allele described by trajectory i is given by the inferred frequencies $q^1_{i'}(t_{i_k})$, here denoted informally by $q^1(t_k)$. Under our deterministic model, these frequencies are described by the difference equation

$$q^1(t_{k+1}) = \frac{q^1(t_k) e^{\sigma^{\text{eff}}_i(t_k)\Delta t_k}}{1 - q^1(t_k) + q^1(t_k) e^{\sigma^{\text{eff}}_i(t_k)\Delta t_k}}, \tag{4}$$

where $\Delta t_k = t_{k+1} - t_k$. This difference equation describes a family of curves which can be uniquely specified by any given value of $q^1(t_k)$. Here, the most central time point, denoted t_c, was used as a parameter to define a curve approximating the evolution of the allele, finding the value of $q^1(t_c)$ which optimised the likelihood fit between the inferred frequencies, q^1 and the observed frequencies $\hat{q}^1$ across the recorded time points.

1.1.5 Likelihood fitting

Likelihood fitting was carried out using a binomal fit between the observed and inferred frequencies. Supposing that the sample at time t_k was of depth n_k, the likelihood of observing the frequency $\hat{q}^1(t_k)$

given a real frequency of $q^1(t_k)$ was calculated as

$$\mathcal{L}(q^1(t_k)) = P(\hat{q}^1(t_k)|q^1(t_k), n_k) = \binom{n_k}{n_k\hat{q}^1(t_k)} q^1(t_k)^{n_k\hat{q}^1(t_k)}(1-q^1(t_k))^{n_k(1-\hat{q}^1(t_k))}, \tag{5}$$

the log likelihood of the trajectory being given by $L_i = \sum_k \log \mathcal{L}(q^1(t_k))$, and the log likelihood for the system, incorporating all trajectories, being given by $\sum_i L_i$.

1.1.6 Optimisation

Inherent selection coefficients were found to maximise the log likelihood for the system, expressed above, beginning with randomly chosen values $\{\sigma_i\}$ and using a simulated annealing method. Within the optimisation, two parameters are fitted per trajectory, the first being an inherent selection coefficient, σ_i, and the second being a single frequency per trajectory, $q^1_{i'}(t_{i_c})$, where t_{i_c} is the central time point of the trajectory i. However, the optimal value of the second parameter, $q^1_{i'}(t_{i_c})$, giving the maximum likelihood fit between the inferred and observed trajectory frequencies, is determined by the shape of the inferred frequencies, which itself is determined by the set of all the inherent selection coefficients, $\{\sigma_i\}$, as described in Eqs 1-4. The optimisation therefore proceeds on two levels; a top-level optimisation is performed over the values $\{\sigma_i\}$, while for each set of $\{\sigma_i\}$ considered, a lower-level optimisation is performed to fit the inferred and observed frequencies for each trajectory.

1.2 Modifications made to the inference framework

Changes were made to the method, as detailed below.

1.2.1 Consideration of sequence data

Sequence data was considered at the codon level. Relative to the two-allele per locus scenario, this introduces the possibility that multiple trajectories might exist at the same loci. We note that, in Eq. 3, above, if $\sigma_j > 0$, the mutant allele at locus j' confers a selective benefit to the mutant allele at i' related to its overlap with the mutant allele at i', and a selective disadvantage to the mutant allele at i' related to its overlap with the wild-type allele at i'. In the case of multiple alleles at each locus, we obtain a similar result to Eq. 3.

We consider a general multi-allele-per-locus system. As described in the main text, mutant alleles at a given locus were identified with respect to the wild-type codon at the same locus. Similarly, selection coefficients of trajectories were defined relative to the fitness of the wild-type. We label the alleles at i' and j' as $\{0, 1, 2, \dots\}$, denoting the wild-type as 0. If the trajectory j describes the evolution of the allele $b \in \{0, 1, 2, \dots\}$ at locus j', we can then write its inherent selection coefficient as a difference of Malthusian fitnesses, $\sigma_j = f^b_{j'} - f^0_{j'}$. The effect that the allele b has on the evolution of the allele $a \in \{0, 1, 2, \dots\}$ at locus i', described by the trajectory i can be written as the sum of two components. Firstly, to the extent that sequences with allele a at locus i' also have the allele b at locus j', selection on b contributes towards the evolution of the allele a. Secondly, to the extent that sequences with an allele other than a at locus i' have the allele b at locus j', selection on b contributes against the evolution of the allele a. We thus obtain

$$\sigma_{ij} = \sigma_j \left[\frac{\hat{q}^{ab}}{\sum_d \hat{q}^{ad}} - \left(\frac{\sum_{c\neq a} \hat{q}^{cb}}{\sum_{c\neq a}\sum_d \hat{q}^{cd}} \right) \right], \tag{6}$$

or alternatively, noting that $\sum_d \hat{q}^{ad}_{i'j'} = \hat{q}^a_{i'}$,

$$\sigma_{ij} = \sigma_j \left[\frac{\hat{q}^{ab}}{q^a} - \left(\frac{\sum_{c\neq a} \hat{q}^{cb}}{1 - \hat{q}^a} \right) \right]. \tag{7}$$

We note that Eq. 3 may be straightforwardly obtained from this result, by setting $a = b = 1$ where $a, b, \in \{0, 1\}$. Our result does not assume that the two trajectories in question are at different loci. Trajectories describing alleles at the same locus fit into our model in a natural way, noting that no haplotype can have differing alleles at the same locus, so that, for example, $q^{ab}_{i'i'} = 0$ for $a \neq b$.

1.2.2 Extended observations

As described in a previous publication [1], extended observations were added to the observed trajectories, representing the non-observation of the mutant allele before the start of each trajectory $(q^a(t_k)) = 0)$, and observations of death or fixation ($q^a(t_k) = 0$ or $q^a(t_k) = 1$ respectively) at the end of each trajectory; this process ensures that the optimisation process takes full account of the fate of each trajectory.

Here, a total of 18 extended observations were added to the end of each trajectory, representing three years of absence of the polymorphism from the population. Sample sizes for extended observations were defined as the sample sizes from appropriate time points. Extended observations before the first sampling point were assigned sample sizes equal to the number of sequences in the first sample, while extended observations after the final sampling point were assigned sample sizes equal to the number of sequences in the last sample. No extended observations were added to the end of trajectories that were polymorphic at the final time of sampling.

1.2.3 Non-polymorphic observations

The formula of Eq. 6 for the effect of polymorphism j on polymorphism i at time t breaks down when $\hat{q}^a(t) = 0$. In such cases, approximations for σ_i^{eff} were made. For extended observations before the beginning of a trajectory, where no information was available about linkage disequilibrium, $\sigma_{ij}(t)$ was assumed to be zero for all j, giving $\sigma_i^{\text{eff}}(t) = \sigma_i$. For extended observations after the final point of a trajectory, $\sigma_i^{\text{eff}}(t)$ was set to the equivalent value for the last observed time of polymorphism; $\sigma_i^{\text{eff}}(t) = \sigma_i^{\text{eff}}(t_{\text{last}})$. For a non-polymorphic observation at some time t_k in the middle of a trajectory i, then where t_{k_1} and t_{k_2} were the times of the last polymorphic observation of i before t_k and the first polymorphic observation of i after t_k, the effective selection coefficient was set as $\sigma_i^{\text{eff}}(t_k) = [\sigma_i^{\text{eff}}(t_{k_1}) + \sigma_i^{\text{eff}}(t_{k_2})]/2$. For extended observations, where a frequency lay outside the interval [0.025,0.975], the effective selection coefficient was set to zero (see next section on frequency cutoffs).

1.2.4 Exclusion of low-frequency trajectories

Previously, trajectories with low maximum frequencies were excluded from the optimisation, removing trajectories with maximum frequency less than a cutoff of $q_{\sigma_i} = \min\{1/N\sigma_i, 0.1\}$, such that only trajectories in which selection played a more significant role than drift were evaluated under the deterministic framework [2]. In the case of influenza, the population size N is unknown, therefore a more heuristic cutoff was applied. Based upon data from simulations (see below), the maximum frequency cutoff was set for all trajectories to a value $q_{\text{cut}} = 0.025$, equal to a frequency of 2.5% This value was set to be low, in order to best maintain accuracy, while accounting for potential sequencing error, and for the existence of extensive polymorphism in the influenza virus among samples within an individual patient. Given this cutoff, in a sample of size 41 or greater, any trajectory had to result from the observation of a polymorphism in at least two sequences in order to be included in the optimisation. In simulations conducted with a large sample size, excluding trajectories with maximal frequencies less than 2.5% had very little negative impact on the accuracy of the inferred selection coefficients (see section 1.3.1). In test optimisations carried out on the influenza data, a cutoff of 0.05 led to a worse fit between observed and inferred frequencies than did a cutoff of 0.025. Subsequent to these calculations being carried out, an estimate that for influenza, $N\sigma = 100$ was published [3]; if this is correct, our cutoff is a conservative estimate.

1.2.5 Constraining the fitting of deterministic trajectories

In another change to the previously published method, in the fitting of a deterministic curve $q^a(t)$ to the observed frequencies $\hat{q}^a(t)$, a minimum value for the parameter $q^a(t_c)$ was imposed, equal to one tenth of the maximal frequency cutoff q_{cut}. This fixed an artefact of the previous code, in which a trajectory which reached a frequency substantially above the cutoff before dying out was assigned a large positive selection coefficient, improving the likelihood of another trajectory through linkage disequilibrium, but in which the same trajectory was assigned an extremely small value of $q^a(t_c)$, giving a deterministic curve essentially equal to zero, avoiding the likelihood penalty of assigning high positive selection to a non-fixing trajectory.

1.2.6 Midpoint approximation for effective selection coefficients

As described above, in the previously published method, calculation of deterministic trajectories was based upon the effective selection coefficients $\sigma_i^{\text{eff}}(t)$, the effective selection calculated for time t_k was used to propagate the deterministic trajectory up to the next sampling time t_{k+1}:

$$q^a(t_{k+1}) = \frac{q^a(t_k) e^{\sigma_i^{\text{eff}}(t_k)\Delta_k}}{1 - q^a(t_k) + q^a(t_k) e^{\sigma_i^{\text{eff}}(t_k)\Delta_k}}, \tag{8}$$

where $\Delta_k = t_{k+1} - t_k$. Where the change in $\sigma_i^{\text{eff}}(t)$ is large between time points, this can be inaccurate. The deterministic trajectory was therefore propagated using a midpoint approximation, which calculated an estimate of the mean selection over the time interval

$$q^a(t_{k+1}) = \frac{q^a(t_k) e^{\frac{1}{2}(\sigma_i^{\text{eff}}(t_k)+\sigma_i^{\text{eff}}(t_{k+1}))\Delta_k}}{1 - q^a(t_k) + q^a(t_k) e^{\frac{1}{2}(\sigma_i^{\text{eff}}(t_k)+\sigma_i^{\text{eff}}(t_{k+1}))\Delta_k}}. \tag{9}$$

Figure S1 shows trajectories inferred for an example observation under the old and new approaches.

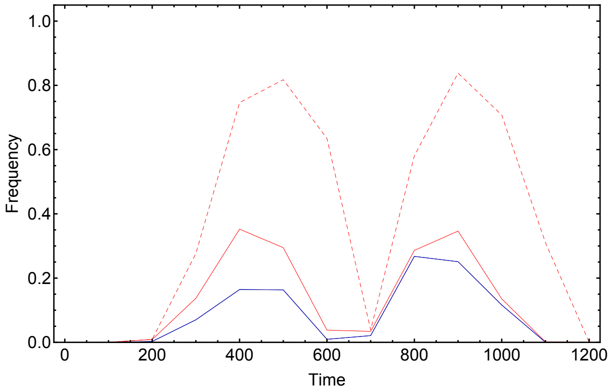

Figure S1. Use of a midpoint approximation improved the inferred trajectories. Illustration of maximum likelihood trajectories inferred using correct selection coefficients, with and without the midpoint approximation. Observed frequencies (blue line) are calculated from a simulation, with sampling time step $dt_s = 100$. The deterministic curve inferred without the midpoint approximation (dotted red line) propagates the trajectory according to the values $\sigma_i^{\text{eff}}(t_k)$, leading to significant errors when the effective selection changes over the time interval. The curve inferred with the approximation (solid red line) propagates the trajectory according to the values $\frac{1}{2}(\sigma_i^{\text{eff}}(t_k) + \sigma_i^{\text{eff}}(t_{k+1}))$, leading to a greatly improved, if not perfect fit, the sparse sampling still producing some error.

1.2.7 Accounting for sampling in the fitting of deterministic trajectories

In the previously published method, allele frequencies were calculated from samples, each of multiple individuals, sampled from the population at a single point in time. In this study, our data consisted of multiple individual sequences, each associated with a sampling time, such that observed frequencies were calculated from sequences collected within a set of overlapping time windows. To evaluate the effect of this, the inference method was applied to trajectories generated by applying a similar sampling process to a population generated using a Wright-Fisher simulation. Given a simulated population, samples from sets of some number n_c of successive time points were pooled, with sequences from n_c consecutive time points being used to describe polymorphism and two-locus haplotype frequencies. Next, an inference was carried out, using the correct inherent selection coefficients from the simulation to infer trajectories. Carrying out this procedure, if the time between samples was sufficiently great, a substantial error was seen in the inferred trajectories, despite having correct values of σ_i, as seen in Figure S2. Upon subsequent optimisation, this error in inferred trajectories led to significant deviations in the inferred σ_i away from the correct values.

In order to account for sampling, a change was made to the inference procedure. During the optimisation of the deterministic curve $q^a(t)$ to fit the observations $\hat{q}^a(t)$, a map was applied to $q^a(t)$, modelling the sampling process via which the $\hat{q}^a(t)$ were generated. Following the calculation of a set of inferred frequencies $q^a(t_k), k = 1, \ldots, K$, the mapped values $q^a(t_k)^*$ were calculated.

$$q^a(t_k) \to q^a(t_k)^* = \frac{1}{n_c} \sum_{l=-n_c}^{n_c} q^a(t_{k+l}), \tag{10}$$

Here, values of $q^a(t_k)$ for $k < 1$ were defined to equal $q^a(t_1)$. Values of $q^a(t_k)$ for $k > K$ were defined to equal $q^a(t_K)$. The mapped frequencies $q^a(t_k)^*$ were then evaluated for their fit to the observed frequencies $\hat{q}^a(t_k)$. Use of this correction led to a substantial improvement in the fit between trajectories (Figure S2 is illustrative).

1.2.8 Binomial fitting model

As for the original method, a binomial sampling model was used to calculate the likelihood of inferred frequencies for each trajectory. This represents an approximation in the case where multiple polymorphic alleles were observed at the same locus. However, it allowed for the fitting between frequencies to be conducted independently for each trajectory, speeding up the optimisation process.

1.3 Assessment of the inference method using simulated data

In order to gain a better understanding of the behaviour of the inference method under different conditions, inferences were made from a set of simulated populations. We first note that our simulations attempt to reproduce, to an extent, the global evolution of the influenza virus, rather than the growth of individual viruses. These two have differing dynamics, the global population comprising viruses from hundreds of thousands or millions of individual infections. For example, the distribution of the selection coefficients of mutations within individual viruses, relative to a population of viruses in a single host, may not equal the distribution of selection coefficients of mutations seen globally, relative to the global viral population. Similarly, the rate at which mutations in the population of influenza viruses reach frequencies large enough to be observed in the global population is not necessarily equal to the mutation rate in individual viruses. Simulations were performed based upon parameters which gave allele frequency trajectories that were, on qualitative inspection, a reasonable approximation to those seen in the influenza data.

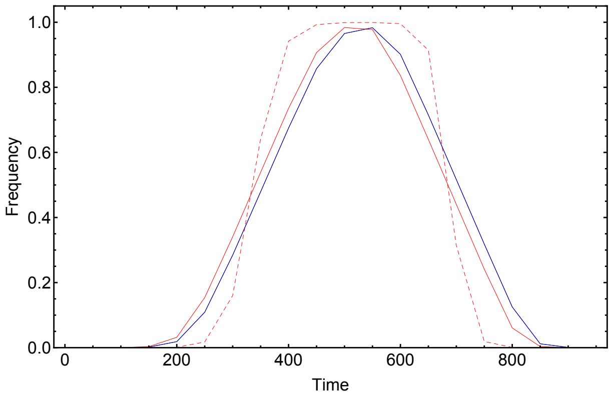

Figure S2. Including a sampling correction improved the inference.
Maximum likelihood trajectories inferred with and without the sampling correction, compared to the observed trajectory (blue line). The curve inferred without sampling correction (dashed red line) propagates the trajectory according to the correct values of $\sigma_i^{\mathrm{eff}}(t_k)$. Sampling, however, has led to a spreading out of the observed frequencies, such that the inferred frequencies give a poor fit to the observation. When sampling is taken into account (solid red line), the sampled curve is much more closely reproduced (albeit with a slight displacement resulting from the time-step between sample points). Frequency data was taken from a Wright-Fisher simulation. Here $n_c = 5$, and $dt_s = 50$ generations.

Wright-Fisher simulations were carried out for populations of size N individuals, each individual containing $L = 5 \times 10^3$ binary loci $\in \{0, 1\}$, set to zero in the first instance, with a mutation rate $\mu = 0.001$ per locus per generation, and selection coefficients on the allele 1 (defined as $f_1 - f_0$, where f_i is the Malthusian fitness of the allele i) being set to a random variable $c\sigma$, where c was set to either 1 or -1 with equal probability, and σ was drawn from an exponential distribution with mean 0.01. Each simulation was run for 10000 generations. Samples from the population were taken every dt_s generations, each of depth n_s. In accordance with the sampling procedure described above, samples were pooled across seven successive sample times ($n_c = 7$). This matched the value used for the real data (where sequences spanning 360 days collected every 60 days), and gave an effective sample of size $7n_s$. Selection coefficients were inferred by running 5×10^5 iterations of a Monte-Carlo method, using a maximal frequency cutoff q_{cut}, taking the final σ_i values as output. Inferred selection coefficients were then compared to the correct coefficients used for the simulation.

1.3.1 High-level sampling

The method was shown to accurately reproduce selection coefficients with a maximum frequency cutoff up to 0.025. Simulations performed at a high level of sampling, with $dt_s = 5$ and $n_s = 10^3$, verified the performance of the method in reproducing selection coefficients under favourable sampling conditions. Optimisations with a population size $N = 10^6$ were used to evaluate the effect of varying maximum frequency cutoffs. Optimisations were carried out for populations generated using a Wright-Fisher process with maximum frequency cutoffs $q_{\mathrm{cut}} \in \{0.0025, 0.005, 0.01, 0.025, 0.05, 0.1\}$. For frequency cutoffs up to and including $q_{\mathrm{cut}} = 0.025$, a very good correlation between inferred and correct σ_i values was observed, falling off for higher cutoffs (Figure S3). This cutoff was applied for subsequent test optimisations, and to the influenza data.

To evaluate the effect of differing population size, populations were simulated as described above, with

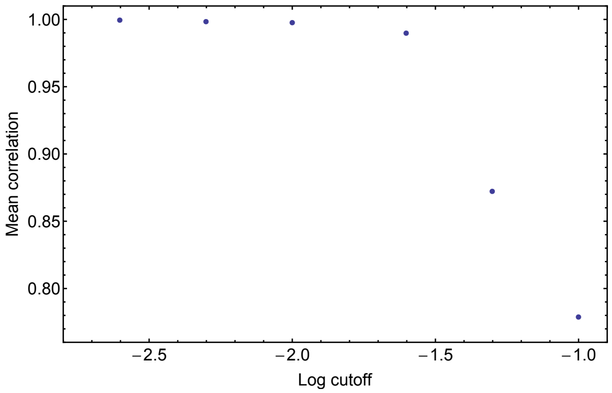

Figure S3. The effect of removing low-frequency trajectories.
Correlation between real and inferred selection coefficients for the example inferences from simulated populations with $N = 10^6$; and a variety of maximum frequency cutoffs. The mean correlation identified with a cutoff of 0.025 was 0.989. The log scale is to base 10.

population sizes $N \in \{10^3, 10^4, 10^5, 10^6\}$, lower population sizes featuring a greater number of trajectories under negative selection, but also greater noise through genetic drift. Twenty simulations, and subsequent inferences (starting from the correct σ_i), were carried out at each N; scatter plots showed a good overall correlation between inferred and correct values of σ_i, increasing with larger N (Figure S4).

1.3.2 Low-level sampling

Simulations performed at lower levels of sampling, with $dt_s = 50$ and $n_s \in \{1, 3, 7, 14, 21, 28, 42, 56\}$ showed that, for population sizes of 10^4 or greater, the method gave reasonably good correlations between real and inferred selection coefficients at sample depths with an effective sample size of 49 or larger. Twenty simulations were performed for each population size and sample depth, inferences starting from randomly generated σ_i in each case. Given an effective sample size of 49, mean correlations were 0.33, 0.50, 0.53, and 0.57 with $N = 10^3$, 10^4, 10^5, and 10^6 respectively. At an effective sample size of 196, the equivalent values were 0.37, 0.56, 0.60, and 0.69 (Figure S5). The distributions of inferred selection coefficients were a reasonable match to the correct distributions (Figure S6).

1.4 Calling of trajectories from aligned RNA sequences

As described in the main text, aligned RNA sequences were matched with their associated dates, recorded in the NCBI influenza virus resource [4]. Beginning at 29th June 1968, samples of sequences were taken every 60 days, each sample comprising all sequences recorded within 180 days of the sample point. The frequency of each codon was measured as a fraction of the unambiguous codons (consisting of unambiguous callings of nucleotides A, C, G, and T) at its respective locus within the population, the sample size from which the codon was drawn being recorded as the number of unambiguous codons at the same locus. A new trajectory was initiated upon the first observation of a mutant codon at a particular locus, the mutant codon being defined with respect to the current wild-type codon at that locus. Determination of the end points of trajectories was carried out in a manner compensating for the finite sample size. If a mutant codon was observed in a sample as having fixed or died, subsequent samples were examined for similar events. If a mutant codon was seen to have fixed or died in eight consecutive observations, the end of the trajectory was defined as the first of those eight observation points. The fixation or death of trajectories was calculated based on the cutoff frequency, q_{cut}, derived earlier; the experimental

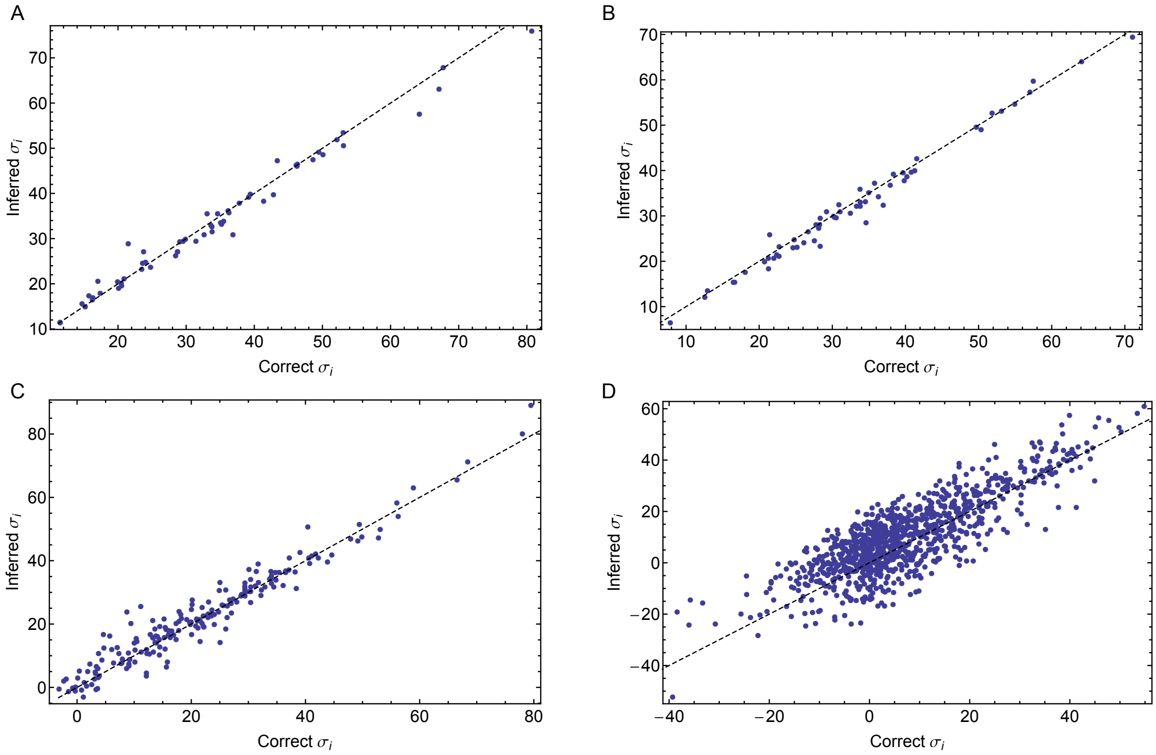

Figure S4. Statistics collected from simulations with high-level sampling. Correlation between real and inferred selection coefficients for example inferences from simulated populations with **(A)** $N = 10^6$; **(B)** $N = 10^5$; **(C)** $N = 10^4$; **(D)** $N = 10^3$. Correlations between datasets presented here are 0.992, 0.992, 0.968, and 0.842, compared to mean values across 20 simulations of 0.989, 0.992, 0.967, and 0.842. The dotted black line shows the line of perfect match between selection coefficients. Selection coefficients are displayed in units of 10^{-3}gen^{-1}.

use of an alternative cutoff is described in section 2.4, later. Frequencies up to and including the end point defined the trajectory of the mutant codon, frequencies subsequent to the end point being discarded.

1.5 Details of the optimisation routine

Optimisations were carried out using an initial simulated annealing process followed by MCMC. Each of these proceeded in a series of iterations. In each iteration, a random change was made to a single trajectory selection coefficient, σ_i. Next, the resulting total log likelihood of the system was calculated, optimising the parameters $q_i^1(t_{i_c})$ for each trajectory given the inherent selection coefficients. The change in log likelihood, ΔL, from the previous iteration, was measured. If ΔL was greater than zero, the change in σ_i was accepted, while if ΔL was less than zero, the change was accepted with probability $e^{\beta \Delta L}$ for some parameter β. In the case $\Delta L = 0$, the change in σ_i was accepted if the magnitude of σ_i decreased, restraining the size of any coefficients which did not affect the overall likelihood.

Given a set of trajectories, an initial set of 400 simulated annealing calculation were run, from random starting points, increasing the parameter β from 0.01 to 0.05 in 2.5×10^5 iterations. The optimisations

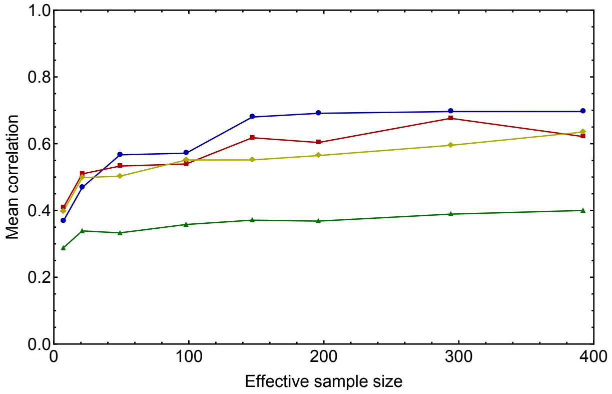

Figure S5. Statistics collected from simulations with low-level sampling.
Correlation between real and inferred selection coefficients for example inferences from simulated populations with $N = 10^6$ (blue circles), $N = 10^5$ (red squares), $N = 10^4$ (yellow diamonds), $N = 10^3$ (green triangles). Displayed correlations are averaged over those obtained from 20 simulations in each case.

with the top 200 log likelihoods were then run for another 2.5×10^5 iterations, increasing β from 0.05 to 0.25 by the end of each run. Following this, the optimisations with the top 100 log likelihoods were then run for another 5×10^5 iterations, increasing β from 0.25 to 1 by the end of each run. Finally, the optimisations with the top 50 log likelihoods were run for a further 2×10^6 iterations, retaining $\beta = 1$. Values of σ_i from the end of each optimisation were taken from each of these 50 calculations. Results from the calculation giving the optimal log likelihood were used for further analysis, the results from the next nine best optimisations being used to assess consistency between optimisations.

2 Consistency of results obtained from the influenza dataset

2.1 Inferred values within a single optimisation

To evaluate convergence within optimisations, selection coefficients inferred after 10^6 iterations of the final optimisation runs were compared to those obtained after the full 2×10^6 iterations. Our results suggested that a good level of convergence had been reached. Eight optimisations out of the fifty calculated were in the top ten both before and after the additional iterations were run; comparing results from these, the mean change in a selection coefficient over the second 10^6 iterations was 0.783 (Figure S7 is representative), producing a mean change in an inferred frequency of 1.7×10^{-3}. Across these optimisations, the mean value of the largest change in an inferred frequency resulting from the extra 10^6 iterations was 0.046.

2.2 Comparison of differences between observed and inferred frequencies

For the maximum likelihood calculation, the mean absolute difference between an observed frequency, $\hat{q}_i(t_k)$, and the corresponding inferred frequency, $q_i(t_k)$ was equal to 0.0497. Absolute frequency differences inferred for synonymous trajectories were fractionally smaller than those inferred for non-synonymous trajectories (Figure S8A). The mean differences across trajectories were 0.0485 and 0.0516 respectively. By comparison, the mean absolute frequency difference for the trajectory shown in Figure 1 of the main

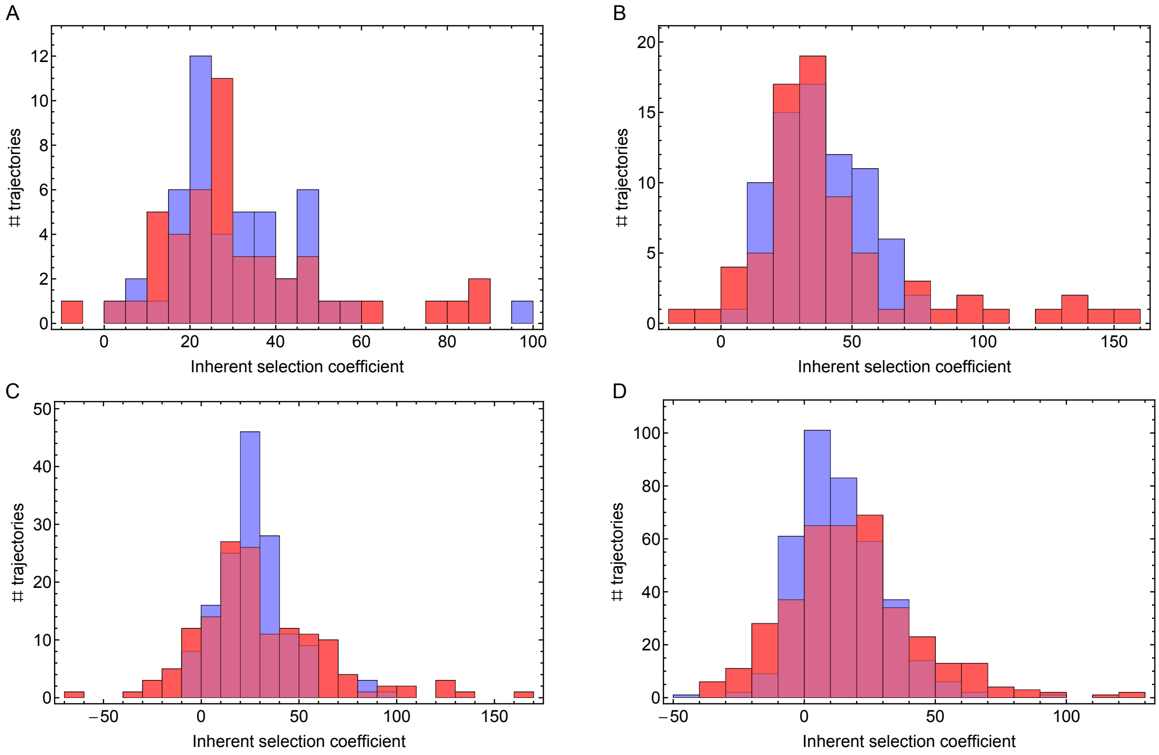

Figure S6. Comparison of input vs. inferred selection coefficients derived from data with low-level sampling.
Histograms showing the distribution of correct (blue) and inferred (red) selection coefficients from representative inferences made with an effective sample size of 196, sampling window $n_c = 7$, and $dt_s = 50$ for simulations of populations of size **(A)** $N = 10^6$; **(B)** $N = 10^5$; **(C)** $N = 10^4$; **(D)** $N = 10^3$. Representative examples of inferred distributions are shown, chosen on the basis of Kolmogorov-Smirnov tests measuring the similarity of the inferred and correct distributions. Overlaps between distributions are shown in pink. Selection coefficients are displayed in units of 10^{-3}gen^{-1}.

text was 0.137. As noted in the main text, differences were substantially larger for the unlinked method than for the linked method. A comparison of frequency differences for the two methods is shown in Figure S8B.

2.3 Inferred values between optimisations

2.3.1 Inferred selection coefficients

Comparison between optimisations of inferred selection coefficients showed a reasonable correlation between results, albeit with some differences in values. Closer observation suggested that the majority of differences occurred for trajectories sampled at earlier time points, for which the number of sequences in a sample was generally lower. Guided by the results obtained from simulations, a cutoff was made at the time point beyond which all samples contained at least 50 sequences, namely that centred on 5th December 1995. Trajectories were then divided into two subsets, comprising those which began at or beyond the identified time point, and those which arose earlier. This divided, in effect, polymorphisms

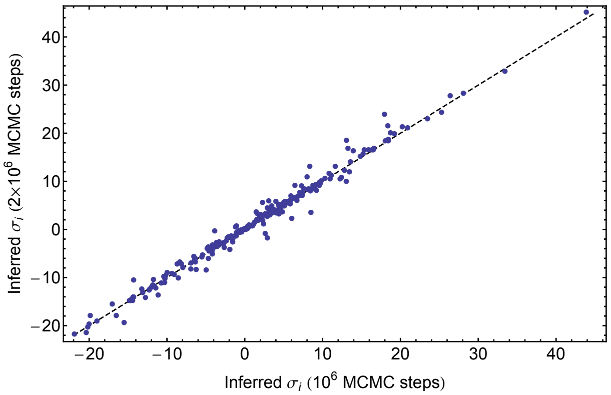

Figure S7. Convergence of the optimisation.
Scatter plot of inherent selection coefficients inferred after 10^6, and 2×10^6 MCMC steps for the optimisation giving the best log likelihoods in each case. The dotted black line indicates equality between selection coefficients. Selection coefficients are displayed in units of 10^{-3}day^{-1}.

which were observed before 1996 from those which were not observed until 1996 or later. The division marked an effective transition between sampling regimes; before the cutoff samples contained a mean of 12 sequences, compared to a mean of 202 sequences per sample after that point.

Pairwise correlations of inferred σ_i values between different optimisations showed consistent results for later trajectories, but less consistent results for earlier trajectories (Figure S9). Across the ten optimisations producing the ten highest log likelihoods, the mean correlation between σ_i values was 0.85 for later trajectories (range 0.78 to 0.89), and 0.49 for earlier trajectories (range 0.31 to 0.66). Inferred selection coefficients for early trajectories included some very high values, likely due to sparse sampling. In the limit, given a sample of depth 1, fixation may be observed as an instantaneous binary transition, associated with arbitrarily high selection; deeper sampling avoids these artefacts.

In our optimisation, selection coefficients were calculated for all polymorphisms, trajectories arising before 1996 affecting those arising later through linkage disequilibrium. However, in calculating statistics of trajectories, only inferences made for trajectories arising in the later time period were used.

2.3.2 Inferred frequencies

Inferred frequencies were highly consistent between optimisations. Differences between inferred frequencies were calculated between each pair of the ten optimisations producing the highest log likelihoods. Examining individual trajectories, a good qualitative fit was seen between inferred frequencies. Calculated across all frequency values for inferred trajectories beginning from 1996 onwards, the mean pairwise difference between inferences for a given frequency was 4.2×10^{-3} (Figure S10).

2.3.3 Statistical properties of selection effects

A high consistency was observed between optimisations for the statistics of different selection properties in separating fixing from dying polymorphisms, described in Figures 3 and 4 of the main text. Values obtained from the optimisation with the best log likelihood were very similar to mean values across the 10 optimisations (Table S1).

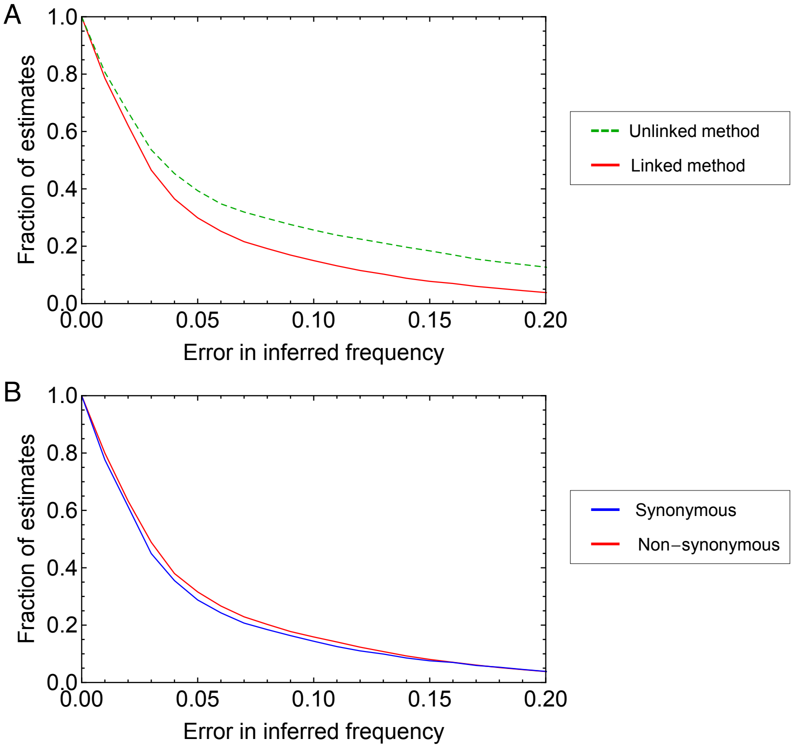

Figure S8. Error profiles for inferred frequencies.
Error values are defined as differences between inferred and observed trajectory frequencies. Curves show the fraction of inferred frequencies with errors greater than a given value. **(A)** Errors in frequencies inferred using the unlinked (green dotted line) and linked (red solid line) methods. For example, for the linked method, just under 15% of inferred trajectory frequencies differed from the observation by more than 0.1, compared to a little over 25% of inferred frequencies from the unlinked method. **(B)** Errors in frequencies inferred by the linked method for synonymous (blue solid line) and non-synonymous (red solid line) trajectories.

2.4 Inferred selection under different methods of trajectory calling

Given finite data from sampling, calling the fixation and death of trajectories is a non-trivial task. For comparison, we performed calculations using an alternative means of identifying trajectories to that noted in the main text. Under this alternative method, trajectories were identified as having fixed if they were observed at a frequency of 1 for eight consecutive sample times, and as having died if they were observed at a frequency of 0 in eight consecutive samples. Inherent selection coefficients were then learnt on the basis of these trajectories.

Comparison of results from each manner of calling trajectories suggested the alternative method to be inferior to that described in the main text. Calculating mean inherent selection coefficients for different regions of the HA protein did not replicate those identified using dN/dS [5], with selection coefficients in the epitope region of the protein fractionally lower than the mean for the protein as a whole (Table S2). Differences between selection coefficients for HA1 and HA2, and for epitope and non-epitope trajectories,

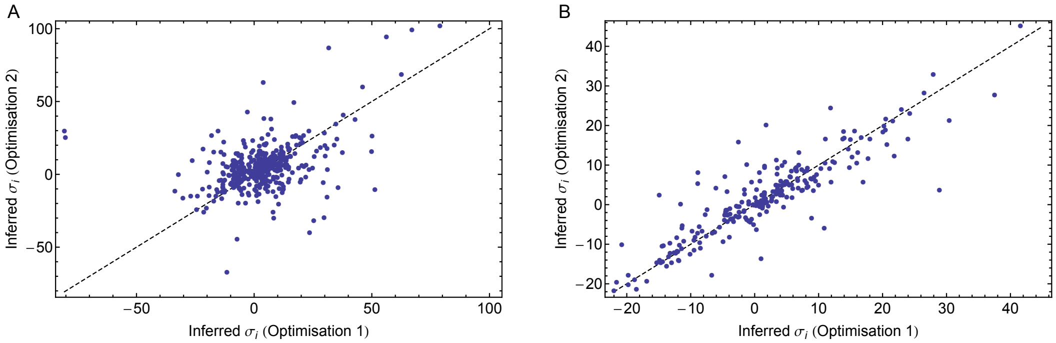

Figure S9. Optimised σ_i for later trajectories were consistent between optimisations. Comparison of inherent selection coefficients inferred for the influenza dataset from the two optimisations producing the highest log likelihoods for: **(A)** Trajectories first observed before 5th December 1995 and **(B)** Trajectories not observed until after 5th December 1995. The correlations between inferred values are 0.46 and 0.89 respectively. The black dotted line shows the line of perfect match between selection coefficients. Selection coefficients are displayed in units of 10^{-3}day^{-1}.

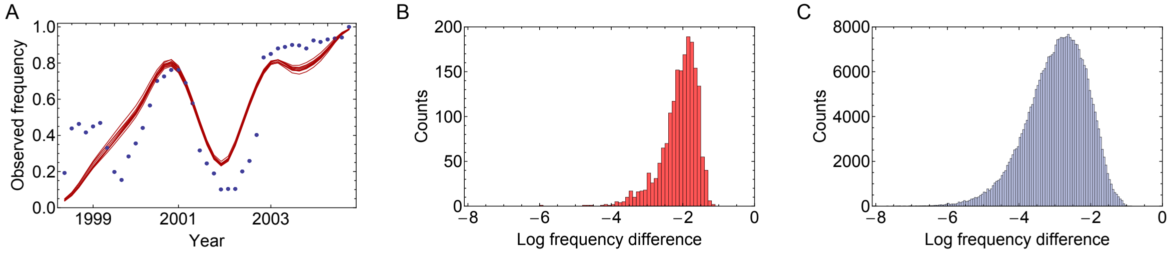

Figure S10. Optimised frequencies were highly consistent between optimisations. **(A)** Inferred trajectories (red) across the ten optimisations giving the best final log likelihoods for the observed trajectory (blue) shown in Figure 1A of the main text. **(B)** Histogram of differences between the inferred trajectories shown for the trajectory of A. The mean error between inferred frequencies is 1.3×10^{-2}. **(C)** Histogram of differences in inferred frequencies for all trajectories starting from 1996 onwards in the influenza dataset across the ten optimisations producing the highest log likelihoods. The mean error between inferred frequencies is 4.2×10^{-3}.

were not significant under a Kolmogorov-Smirnov test. Notwithstanding this, statistics of selection were calculated from the alternative trajectory inferences, and the importance of these statistics for separating fixing from dying trajectories (under the alternative definition) were calculated. Each of the components σ_i^{b}, $\bar{\sigma}_i^{\mathrm{p}}$, and $\bar{\sigma}_i^{\mathrm{t}}$ was found to have a stronger positive influence upon the fate of a trajectory, though the relative importance of inherent selection was preserved. In this alternative set of trajectories, the result of interference effects outweighing inherent selection was preserved (Figure S11).

Table S1. Consistency of statistics between optimisations. Accuracy measurements for selection statistics in separating fixing from dying polymorphisms. The first row gives values described in the main text, which were derived from the optimisation giving the best log likelihood. Subsequent rows give mean values and standard deviations across the best 10 optimisations.

Optimisation	Statistic				
	σ_i	σ_i^{b}	$\bar{\sigma}_i^{\mathrm{p}}$	$\bar{\sigma}_i^{\mathrm{t}}$	$\bar{\sigma}_i^{\mathrm{eff}}$
Best optimisation	0.70	0.64	0.70	0.82	0.96
Mean of 10 optimisations	0.69	0.64	0.71	0.83	0.97
Standard deviation	1.0×10^{-2}	7.5×10^{-3}	6.8×10^{-3}	8.3×10^{-3}	1.7×10^{-3}

Table S2. Mean inherent selection coefficients for regions of the HA gene, inferred from an alternative calling of trajectories. Mean values of σ_i inferred for non-synonymous trajectories within the HA1 and HA2 parts of the gene, and for epitope and non-epitope residues, considering both fixing and dying trajectories.

Protein sites	Mean inherent selection		
	All trajectories	Fixing trajectories	Dying trajectories
HA	-0.72	3.02	-1.24
HA1	-0.54	3.06	-1.07
HA2	-1.64	2.68	-1.93
Epitopes	-0.90	3.28	-1.88
Non-epitopes	-0.51	1.86	-0.63

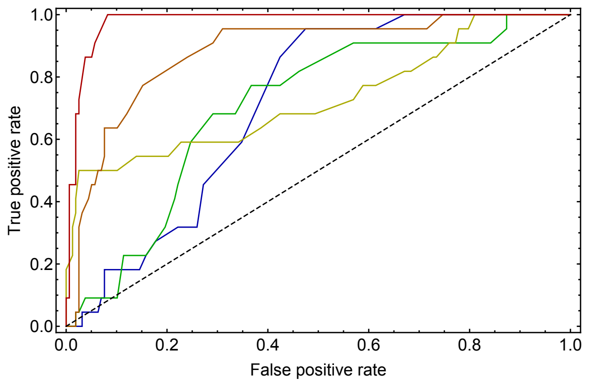

Figure S11. Ability of retrospective measurements of selection to divide fixing from non-fixing trajectories, with trajectories called using the alternative method. Statistics are derived from inherent selection coefficients learnt from the alternative set of trajectories. Ability of retrospective measurements of selection to divide fixing from non-fixing trajectories. Values are shown for the inherent selection coefficient, σ_i (blue), the initial background fitness, σ_i^{b} (green), the mean post-emergence interference, $\bar{\sigma}_i^{\mathrm{p}}$ (yellow), the total mean interference, $\bar{\sigma}_i^{\mathrm{t}}$ (orange), and the mean effective selection coefficient, $\bar{\sigma}_i^{\mathrm{eff}}$ (red) of a trajectory. The black dotted line indicates a null expectation, describing a hypothetical method with no power to divide fixing from non-fixing trajectories.

3 Further calculations performed on the influenza dataset

3.1 Number of trajectories influencing the effective selection coefficient $\sigma_i^{\mathrm{eff}}(t)$

As noted in the main text, identifying simple explanations for the fixation or death of one trajectory in terms of linkage disequilibrium with another is a difficult exercise, with many trajectories influencing each other. In the mean, a trajectory was influenced by a total of 70 other non-synonymous trajectories at some point in its evolution. Many of these had a significant effect on the effective selection coefficient. In the mean, at any given point in time, 15 trajectories had an effect of more than 0.5 on the effective selection coefficient, 21 had an effect greater than 0.25, and 29 had an influence greater than 0.1.

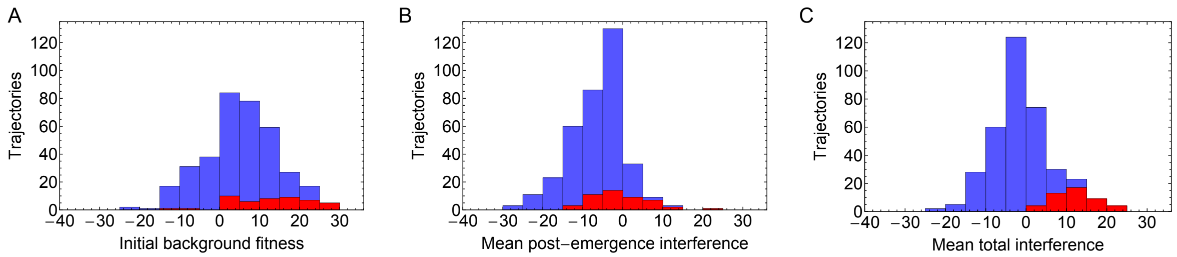

Figure S12. Breakdown of selection and interference effects in synonymous trajectories. Histograms show the distribution of values obtained for synonymous trajectories, obtained from the first method of calling trajectories, which died (blue), and which proceeded to fixation (red) for **(A)** Initial background fitness σ_i^b; **(B)** Mean post-emergence interference $\bar{\sigma}_i^p$; **(C)** Mean total interference $\bar{\sigma}_i^t$. Distributions are calculated across a total of 359 trajectories.

3.2 Components of selection for synonymous trajectories

Histograms detailing the values obtained for components of selection for fixing and dying synonymous trajectories are shown in Figure S12.

3.3 Inherent selection and interference

A plot was made showing the inherent selection coefficients inferred from the influenza data versus the calculated mean interference experienced by each polymorphism (Figure S13). A negative correlation between these values was seen, with slope -0.51 and correlation coefficient -0.57. Such a correlation was also seen in a simple population simulation, suggesting that the result may reflect an underlying property of asexual systems. A Wright-Fisher simulation was run with a large population size, $N = 10^6$, 50 binary loci, mutation rate 5×10^{-8}, and inherent selection coefficients taken from an exponential distribution with mean 0.01. The simulation was run for 10^6 generations, with snapshots of the frequency of each allele, and of the effective selection coefficient it experienced, taken every 100 generations. Mean effective selection coefficients were calculated over the period in which an allele had a frequency within the interval [0.025, 0.975]. Upon reaching a frequency of 0.975, an allele frequency was set to zero, the inherent selection coefficient of its mutant allele being replaced by another draw from the random distribution. Results from the simulation (which were directly calculated, rather than being inferred) matched the pattern of negative correlation seen in our inference from the influenza data. A strong negative correlation with coefficient -0.95 was identified; the best fit by linear regression was slightly steeper than that seen for the influenza data, at -0.69.

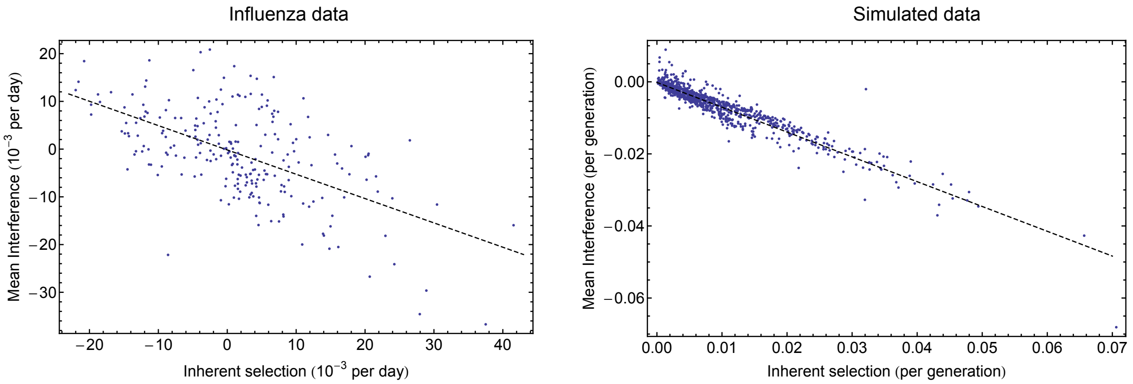

Figure S13. Negative correlation between inherent selection and interference. Results from the influenza data (left) show inferred inherent selection coefficients, σ_i, and mean total interferences, $\bar{\sigma}_i^t$. The correlation coefficient between these values is -0.57; the best fit by linear regression has slope -0.51. Results from simulation data (right) were calculated directly from known selection coefficients. The correlation coefficient between values is -0.95; the best fit by linear regression has slope -0.69. Black dotted lines show best fits by linear regression.

3.4 Mean selection coefficients from the unlinked method and dN/dS

In the main text, a comparison was made between mean selection coefficients identified for regions of the HA gene, and values of dN/dS calculated for the same regions in an earlier work [5]. It was shown that mean selection coefficients match the ordering of epitope > HA1 > HA2 identified by Wolf et al; this ordering was preserved among subsets of trajectories which fixed or dies (see Table 1 of the main text

Table S3. Mean inherent selection coefficients for regions of the HA gene, obtained from the unlinked method. Mean values of σ_i inferred for non-synonymous trajectories within the HA1 and HA2 parts of the gene, and for epitope and non-epitope residues, considering both fixing and dying trajectories

Protein sites	Mean inherent selection		
	All trajectories	Fixing trajectories	Dying trajectories
HA	3.45	10.40	2.11
HA1	3.48	10.96	2.03
HA2	3.28	7.67	2.48
Epitopes	3.77	11.43	1.62
Non-epitopes	3.09	7.83	2.58

and accompanying discussion).

The calculation performed in the main text was repeated using selection coefficients inferred using the unlinked method. For all trajectories, and those which fixed, the order epitope > HA1 > HA2 was preserved, though this order was reversed for trajectories which died (Table S3). Use of a Kolmogorov-Smirnov test, which for the linked method identified significant differences between selection coefficients inferred for different protein regions (see main text), did not in this case identify any significant differences between the distributions of selection coefficients.

3.5 Printout of all trajectory fits

In Figures S14 to S16 we show complete inferences for both the linked and unlinked inferences of selection, and for the linked method applied to the alternative set of trajectories considered above. These figures are large, and are not intended to be printed, so are included as separate files.

Figure S14 Frequencies inferred using the unlinked method.
Observed frequencies (blue dots), and inferred frequencies are shown for synonymous (green dashed lines) and non-synonymous (red dashed lines) polymorphisms. For each trajectory, the initial codon is labelled in black, with the mutant codon labelled in green (synonymous) or red (non-synonymous), according to the nature of the substitution, relative to the wild-type codon. Markers for observed frequencies are, in order of trajectory appearance, solid circles, solid squares, solid diamonds, solid triangle up, solid triangle down, open circles, and open squares.

Figure S15 Frequencies inferred using the linked method.
Observed frequencies (blue dots), and inferred frequencies are shown for synonymous (green lines) and non-synonymous (red lines) polymorphisms. For each trajectory, the initial codon is labelled in black, with the mutant codon labelled in green (synonymous) or red (non-synonymous), according to the nature of the substitution, relative to the wild-type codon. Markers for observed frequencies are as for figure S14.

Figure S16 Frequencies inferred applying the linked method to trajectories derived from the alternative calling method.
Observed frequencies (blue dots), and inferred frequencies are shown for synonymous (green lines) and non-synonymous (red lines) polymorphisms. For each trajectory, the initial codon is labelled in black, with the mutant codon labelled in green (synonymous) or red (non-synonymous), according to the nature

of the substitution, relative to the wild-type codon. Markers for observed frequencies are as for figure S14.

References

1. Illingworth CJR, Mustonen V (2011) Distinguishing driver and passenger mutations in an evolutionary history categorized by interference. Genetics 189: 989–1000.

2. Rouzine I, Rodrigo A, Coffin J (2001) Transition between stochastic evolution and deterministic evolution in the presence of selection: general theory and application to virology. Microbiology and molecular biology reviews 65: 151.

3. Strelkowa N, Lässig M (2012) Clonal interference in the evolution of influenza. Genetics 192: 671–82.

4. Bao Y, Bolotov P, Dernovoy D, Kiryutin B (2008) The influenza virus resource at the National Center for Biotechnology Information. Journal of virology 82: 596–601.

5. Wolf YI, Viboud C, Holmes EC, Koonin EV, Lipman DJ (2006) Long intervals of stasis punctuated by bursts of positive selection in the seasonal evolution of influenza A virus. Biol Direct 1: 34.
