# Supplementary material for: Components of Selection in the Evolution of the Influenza Virus: Linkage Effects Beat Inherent Selection
Source: PLoS Pathog. 2012 Dec 27;8(12):e1003091. doi: 10.1371/journal.ppat.1003091 (PMC3531508; doi:10.1371/journal.ppat.1003091)

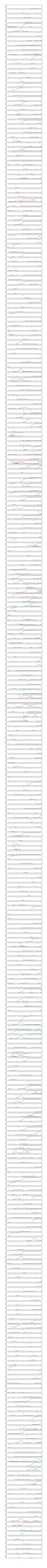

Supplement: Figure S16 — Plots of all observed and inferred trajectories inferred using the linked method with trajectories called using an alternative method. (PDF) [file ppat.1003091.s004.pdf]
